# Interfacial Stabilization of Organic Electrochemical Transistors Conferred Using Polythiophene-Based Conjugated Block Copolymers with a Hydrophobic Coil Design

**DOI:** 10.1021/acsami.4c13197

**Published:** 2024-09-17

**Authors:** Chia-Ying Li, Guo-Hao Jiang, Tomoya Higashihara, Yan-Cheng Lin

**Affiliations:** † Department of Chemical Engineering, National Cheng Kung University, Tainan 70101, Taiwan; ‡ Department of Organic Materials Science, Graduate School of Organic Materials Science, Yamagata University, 4-3-16 Jonan, Yonezawa, Yamagata 992-8510, Japan; § Advanced Research Center for Green Materials Science and Technology, National Taiwan University, Taipei 10617, Taiwan

**Keywords:** electrochemical transistors, poly(3-hexylthiophene), poly(^*n*^butyl acrylate), poly(ethylene oxide), device stability

## Abstract

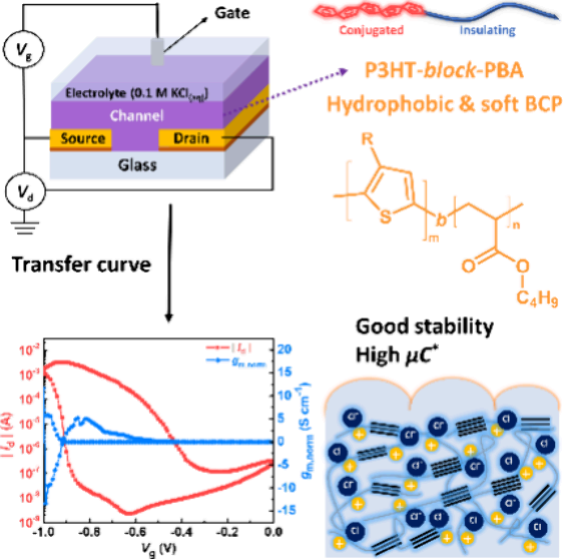

The recent interest in developing low-cost, biocompatible, and lightweight bioelectronic devices has focused on organic electrochemical transistors (OECTs), which have the potential to fulfill these requirements. In this study, three types of poly(3-hexylthiophene) (P3HT)-based block copolymers (BCPs) incorporating different insulating blocks (poly(^*n*^butyl acrylate) (PBA), polystyrene, and poly(ethylene oxide) (PEO)) were synthesized for application in OECTs. The morphological, crystallographic, and electrochemical properties of these BCPs are systematically investigated. Accordingly, P3HT-*b*-PBA demonstrates superior performance in the KCl-based aqueous electrolyte, with a higher product of mobility and capacitance (μC*) at 170 F s^–1^ cm^–1^ V^–1^ than that of the P3HT homopolymer at 58 F s^–1^ cm^–1^ V^–1^. P3HT-*b*-PBA exhibits better stability over 50 ON/OFF switching cycles than do other BCPs and P3HT homopolymers. With regard to the performance in the KPF_6_-based aqueous electrolyte, P3HT-*b*-PBA outperforms with a higher μC* of 9.2 F s^–1^ cm^–1^ V^–1^ than that of 8.6 F s^–1^ cm^–1^ V^–1^ observed from P3HT. Notably, both polymers exhibited almost no decay in device performance over 110 ON/OFF switching cycles. The strongly different performance of polymers in these two electrolytes is due to the side chain’s hydrophobicity and interdigitated lamellar structures, thereby retarding the doping kinetics of the highly hydrated Cl^–^ ions compared with the slightly hydrated PF_6_^–^ ions. Concerning the improved performance of P3HT-*b*-PBA, this is attributed to its soft and hydrophobic backbone. Our morphological and crystallographic analyses reveal that P3HT-*b*-PBA experiences minimal structural disorder when swelled by the electrolyte, maintaining its original structure better than the P3HT homopolymer and the hydrophilic BCP of P3HT-*b*-PEO. The hydrophobic nature of P3HT-*b*-PBA contributes to the stability of its backbone structure, ensuring enhanced capacitance during the operation of the OECT operation. These findings provide reassurance about the stability and performance of P3HT-*b*-PBA in the field of OECT applications. In summary, this study represents the first exploration of P3HT-based BCPs for OECT applications and investigates their structure–performance relationships in mixed ionic–electronic conductors.

## Introduction

In the rapidly advancing field of healthcare today, there is a growing emphasis on preventing and monitoring diseases, especially chronic diseases like diabetes. It requires precise monitoring and effective treatment. In this regard, the application of biosensors has become a novel and up-and-coming area. To make the devices more convenient and widespread, developing low-cost, biocompatible, and lightweight bioelectronic devices is critical. Organic electrochemical transistors (OECTs) have the potential to fulfill the above requirements. It is an electronic device in which the conjugated polymer (CP) directly interfaces with an electrolyte, featuring a gate electrode immersed in it. The CP undergoes electrochemical doping by applying a gate bias, altering its redox state and conductivity. Ions then penetrate the polymer film to neutralize the charge.^1,2^ OECTs utilize ionic and electronic transport to transduce into drain current in electronic devices.^3^ In contrast to organic field-effect transistors (OFETs), which induce carriers through the dielectric effect, OECTs operate in an electrolyte, relying on electrochemical doping/dedoping of CPs. This allows for carrier generation throughout the polymer volume, resulting in high OECT drain currents,^4,5^ and enables OECTs to function at low electrochemical potentials below 1 V.^6,7^ OECTs possess properties suitable for various applications. They show promise in biosensing, detecting metabolites such as dopamine,^8^ glucose,^9^ and cortisol.^10^ In addition, they can be employed in electrophysiological analyses like electrocorticography,^11^ electrocardiogram,^12^ and electrooculography.^13^ OECT devices can also be fabricated on flexible substrates, making them suitable for potential use in wearable devices.^14,15^

Optimizing the OECTs involves addressing several key challenges based on their working principles. First, selecting a suitable CP for OECTs requires careful consideration, emphasizing both good ionic and electronic conductivity. This demand for CP design surpasses that of OFETs.^16,17^ Second, dealing with the slow doping kinetics is important. This slowness is attributed to poor hole mobility at low doping levels (low electrochemical potential) and the slow diffusion of large ions in OECTs, limiting their applicability in scenarios that demand fast responses at high frequencies.^17−19^ Moreover, enhancing the device stability is also necessary. Undesirable side reactions, such as the reduction of oxygen to hydrogen peroxide, contribute to the degradation of CPs.^20,21^ Preventing irreversible redox reactions of CPs at high voltages is imperative.^22^ Swelling caused by hydration and doping can damage the structure of CPs, reducing their mobility and stability during electrochemical doping.^23^ In addition, it is also important to consider the possibility of electrolyte components penetrating the polymer film and reacting with source/drain electrodes (e.g., gold) when a gate bias is applied.^24^ In comparison to an n-type OECT with Na^+^/K^+^ doping to n-type CPs, the effect of chlorination is more serious in a p-type OECT with Cl^–^ doping to p-type CPs.

Several solutions have been proposed for the challenges mentioned above. Regarding CPs design, Wang and co-workers^25^ emphasized that enhancing charge transport involves achieving high backbone coplanarity, which can be accomplished by introducing intramolecular hydrogen bonds. Flagg and co-workers^26^ explored the impact of hydrophilic side chains and crystallinity on OECT performance. Their spectro-electrochemical measurements revealed that polythiophene with hydrophilic side chains exhibited higher conductivity and faster response, indicating improved doping processes. They also discovered that with high crystallinity of CPs, the presence of water destroys the electronic connectivity between the crystalline regions during electrochemical doping. Huang and co-workers^27^ reported that porous polymer films with a large surface area achieve high capacitance and rapid ion intercalation. Sun and co-workers^28^ revealed that the alkyl group content in the carboxyl side chain not only influences the overall hydrophilicity of the polymer but also determines the degree of swelling. Specifically, the propyl variant exhibits the highest hydrophilicity, resulting in excessive swelling during the doping process and a reduction in hole mobility. Conversely, the hydrophobic pentyl spacers restrict swelling, impeding ion injection into the polymer film and complicating the doping process. Therefore, moderate introduction of hydrophobic side chains or spacers is a viable approach for enhancing device stability. To address oxygen reduction, although removing oxygen in electrolytes can mitigate this issue, it is still inconvenient for practical biomedical applications. Zhang and co-workers^29^ designed a polymer glue, consisting of lithium bis(trifluoromethanesulfonyl)imide (LiTFSI) and poly(ethylene oxide) (PEO) as a protective layer to improve the device stability by preventing water and oxygen penetration into a polymer film. In addition, lowering the energy level of CP is also a solution to operate in a low electrochemical window, avoiding oxygen reduction.^30^

In addition to the CP designs mentioned above, the design of conjugated block copolymers presents another viable approach to solving the identified challenges. Wang and co-workers^31^ explored the synthesis of BCPs using varying ratios of poly(^*n*^butyl acrylate) (PBA) and poly(3-hexylthiophene) (P3HT), investigating the relationship between PBA content and OFET performance. They observed that the morphology, crystallinity, and OFET performance of BCP varied with the P3HT-to-PBA ratio. Yang and co-workers^32^ previously highlighted the significance of the insulating block in P3HT-based conjugated BCPs and its impact on properties, particularly in the context of photosynaptic transistors. The structure of BCPs varies with the rigidity of the insulating block. The flexibility of adjusting BCP properties is advantageous given the numerous components and compositions available. Various factors, such as the degree of polymerization, block ratio, Flory–Huggins interaction parameter (χ) between blocks, and annealing conditions, contribute to diverse BCP structures.^33^ For example, Kamp and co-workers synthesized block copolymers of polythiophene and polyethylene glycol, leading to self-assembled one-dimensional assemblies (nanofibers) in selective solvents like water and methanol.^34^ Therefore, the synthesis conditions also play an important role in the structure of a BCP. There are many factors or variables that can be tuned for BCP design. Despite the potential in BCP design, there is currently no reported application of BCPs in OECTs, and the impact of backbone properties remains unexplored. Therefore, it is crucial to explore the principles of BCP design, considering how both structural and morphological properties influence the performance of the components in the construction of the OECTs.

In this study, P3HT-based conjugated BCPs incorporating different insulating segments such as PBA, polystyrene (PS), and PEO were used as the active layers of the OECTs. Atomic force microscopy (AFM), grazing incident wide-angle X-ray scattering (GIXD), and in situ spectro-electrochemical measurements were conducted to analyze the morphology, molecular packing, and doping behavior of these BCPs. For OECT characteristics, threshold voltage (*V*_th_) and OECT’s figure-of-merits (μC*) were primarily used to evaluate their performance. The effects of electrolytes and hydrophobicity of the doping ions on the P3HT and BCP’s performances in the OECT devices were evaluated. Accordingly, KCl and KPF_6_ were applied as the salt in aqueous electrolytes in OECT operations. We found enormously different performances of polymers in these two electrolytes due to their varied doping kinetics regarding the hydrated water molecules and hydrophobicity of the anions. Concerning the structure/performance relationship of the BCPs, the results show that the insulating segment affects the hydrophilicity and rigidity of BCP, which also influence their OECT performance. BCPs with soft and hydrophobic blocks help stabilize their structures, which is beneficial for device stability. While adding the hydrophilic PEO block, the AFM morphology indicated film delamination after swelling, related to its low μC* and poor stability. Moreover, P3HT-*b*-PS, with a relatively rigid PS block, shows higher crystallinity in the as-cast film, but the packing is ruined after swelling, resulting in relatively low OECT performance. This is the first study focusing on the design of conjugated BCPs for OECT applications and the relationship between the structure of BCPs and the improvement of device stability.

## Experimental Section

### Materials

Poly(3-hexylthiophene-2,5-diyl) (P3HT) (UR-P3H001, regioregular, > 99%, *M*_w_ = 50 000–72 000) was purchased from Uni-Region Bio-Tech. Poly(3-hexylthiophene)-*block*-poly(styrene) (P3HT-*b*-PS, *M*_n_ (P3HT) = 10 000, *M*_n_ (PS) = 13 500, *Đ*_M_ = 4.5) was purchased from Polymer Source, Inc. (Quebec, Canada). Poly(3-hexylthiophene)-*block*-poly(^*n*^butyl acrylate) (P3HT-*b*-PBA, *M*_n_ (P3HT) = 6 000, *M*_n_ (PBA) = 6 000, *Đ*_M_ = 1.15) was synthesized according to a reported method using the click reaction between alkynyl-terminated P3HT and azido-terminated PBA.^31^ Poly(3-hexylthiophene)-*block*-poly(ethylene oxide) (P3HT-*b*-PEO, *M*_n_ (P3HT) = 8 130, *M*_n_ (PEO) = 5 000, *Đ*_M_ = 1.45) was synthesized by modifying a reported method using the click reaction between azide-terminated P3HT and alkyne-terminated PEO.^35^ Chloroform (CF, ≥99.8%) and potassium hexafluorophosphate (KPF_6_, ≥99%) were purchased from Sigma-Aldrich. Potassium chloride (KCl, ≥99.0%) was purchased from J.T. Baker.

### Characterization

UV–vis–NIR absorption spectra were obtained by using a JASCO V-770 spectrophotometer. Polymer films were spin-coated onto quartz substrates, and bandgaps (*E*_g_) were calculated from the polymer film’s absorption onset (λ_onset_) using the equation: *E*_g_ (eV) = 1240/λ_onset_. Cyclic voltammetry (CV) was performed with a CHI 6273E electrochemical analyzer in a three-electrode cell system. The working electrode was a polymer-coated ITO glass, the counter electrode was a platinum foil, and Ag/AgNO_3_ served as the reference electrode for the nonaqueous electrolyte, while Ag/AgCl was used for the aqueous electrolyte. The highest occupied molecular orbital (HOMO) levels of the polymer films were determined in 0.1 M tetrabutylammonium perchlorate (TBAP) solution dissolved in dry acetonitrile. HOMO levels (eV) were calculated using onset oxidation potentials (*E*_onset,org_) for the polymers and the redox couple of ferrocene as an internal standard (*E*_ferrocene_^1/2^) with the equation HOMO = −*e*[*E*_onset,org_–*E*_ferrocene_^1/2^ + 4.8]. The lowest unoccupied molecular orbital (LUMO) levels were calculated by using HOMO levels and optical bandgaps. For the assessment of oxidation onset potentials in the aqueous electrolyte (*E*_onset,aq_), CVs were measured using 0.1 M aqueous KCl solution as the electrolyte. Prior to the CV measurement, the electrolyte was degassed by nitrogen purging. Atomic force microscopy (AFM) images of the polymer films were obtained using Multifunctional Scanning Probe Microscopes (Bruker) operated in tapping mode. Contact angle measurements were conducted using video contact angle optima (AST Products, Inc.). Grazing-incidence wide-angle X-ray scattering (GIWAXS) results were obtained at the BL13A1 beamline at the National Synchrotron Radiation Research Center (NSRRC), Taiwan, with an incidence angle of 0.12° and a monochromatic beam wavelength of 1.027 Å. The polymer-coated silicon wafer was utilized for the analysis, employing the same coating parameters as those mentioned earlier.

In situ spectro-electrochemical measurements were conducted using the same setup as CV in a three-electrode system, with a 0.1 M aqueous KCl solution as the electrolyte. The system was fitted inside a quartz cuvette within the UV–vis–NIR absorption spectrophotometer. The three electrodes remained unchanged. Before recording the spectrum, an electrochemical potential was applied until a steady current was achieved (approximately 30 s) to ensure steady-state electrochemical optical behavior. Potentials ranged from −0.2 to 1.2 V, with an increment of 0.1 V. The resulting polymer spectrum was normalized by its maximum absorbance under different biases. Electrochemical impedance spectroscopy (EIS) was performed using the same setup as that of CV, employing a three-electrode system in a 0.1 M aqueous KCl solution as the electrolyte. The working electrode was a polymer-coated ITO glass, following the same coating process. The measurement involved applying an amplitude of 10 mV and scanning between 0.1 Hz and 10 kHz. Capacitance was determined by fitting to the equivalent circuit by using the CHI 6273E software.

### Device Fabrication and Characterization

The OECT device followed a standard top-gate/bottom-contact (TG/BC) architecture with a glass/chromium/gold/BCP configuration. The glass substrate underwent cleaning with 2-propanol and acetone. Chromium (used as an adhesion layer) and gold were subsequently deposited on the glass substrate using a customized mask, with thicknesses of 20 and 40 nm, respectively. The evaporation process had a rate of 0.5 Å s^–1^, and the pressure was maintained below 5 × 10^–6^ Torr. The interdigitated channel had a length (*L*) of 25 μm and a width (*W*) of 9 000 μm (consisting of nine parallel channels, each with *W* = 1000 μm). Polymer solutions, with a concentration of 10 mg mL^–1^ in CF, were heated at 60 °C for 2 h and then spin-coated onto the electrodes at a spin rate of 2000 rpm for 40 s. A partial swiping with CF was done to clean and expose the electrodes. Film thicknesses were measured using an Alpha-Step D300 profilometer.

For the OECT characterization, a 0.1 M aqueous KCl solution served as the electrolyte and was contained in a polydimethylsiloxane (PDMS) well placed on the device. A Ag/AgCl pellet (1.0 × 2.5 mm, A-M SYSTEMS) was used as a gate electrode and immersed in the electrolyte. All characteristics were measured by using a Keithley 4200-SCS semiconductor parameter analyzer, and the device position was securely fixed via vacuum pumping. Transfer curves were generated by applying varying gate voltages (*V*_g_) from 0 to −1.0 V, with a fixed drain voltage (*V*_d_) of −0.5 V and a step size of 0.01 V, with a 1-s delay between each step. Dual sweeping was employed to return the chromatogram to the original state. Transconductance (*g*_m_) was determined using the following equation:
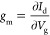
1

To facilitate a better comparison of *g*_m_, the normalized transconductance (*g*_m,norm_) was obtained by using the following equation:
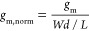
2

The OECT’s figure of merit, μC*, was obtained according to the following equation:
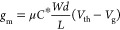
3

The slope of *g*_m_ versus  is the μC* value. Before getting the μC* value, threshold voltage *V*_th_ was beforehand determined by fitting the linear region of *I*_d_^1/2^ vs *V*_g_. The *x*-intercept of the fitting line is the *V*_th_ value. Carrier density (*p*) was calculated by the equation:
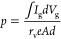
4with ∫*I*_g_*dV*_g_ for the integrated value of the curve *I*_g_ versus *V*_g_, *r*_v_ for the scan rate of the *V*_g_ (10 mV s^–1^), *e* for the elementary charge (1.6 × 10^–19^ C), *A* for the effective gate area (0.07854 cm^2^), and *d* for the channel thickness of each polymer. Output curves were generated by varying *V*_g_ from 0 to −0.6 V with a step size of 0.01 V, with a 1-s interval between each step and a stepped *V*_g_ of 0 to −0.8 V. Dual sweeping was employed to return to the original state. Transient curves were obtained using switched *V*_g_ of 0 and −0.8 V with switch lengths of 15 and 30 s and a fixed *V*_d_ of −0.5 V. Rise time (*t*_r_) and fall time (*t*_f_) were determined through exponential fitting (*y* = *y*_0_ + *A*_1_exp(−*x*/t_1_), where *y* is *I*_d_, *x* is time, and *t*_1_ represents *t*_r_ or *t*_f_). Stability tests were conducted using switched *V*_g_ of 0 and −0.9 V with a switch length of 15 s and a fixed *V*_d_ of −0.1 V, repeated for 10 cycles. Transient gate current analysis at the time domain to obtain mobility was conducted by applying different *I*_g_, and the reciprocal transit time (−1/τ_e_) was determined by linear fitting of d*I*_d_/d*t* vs *I*_g_ curve, and the slope is −1/τ_e_. Then, the mobility was calculated by , where *n* is 9 for nine parallel channels in the interdigit electrode pattern, *L* is the channel length (25 μm), and *V*_d_ is fixed at −0.5 V.^36,37^

## Results and Discussion

### Optical and Electrochemical Properties of BCPs

**Figure 1:**
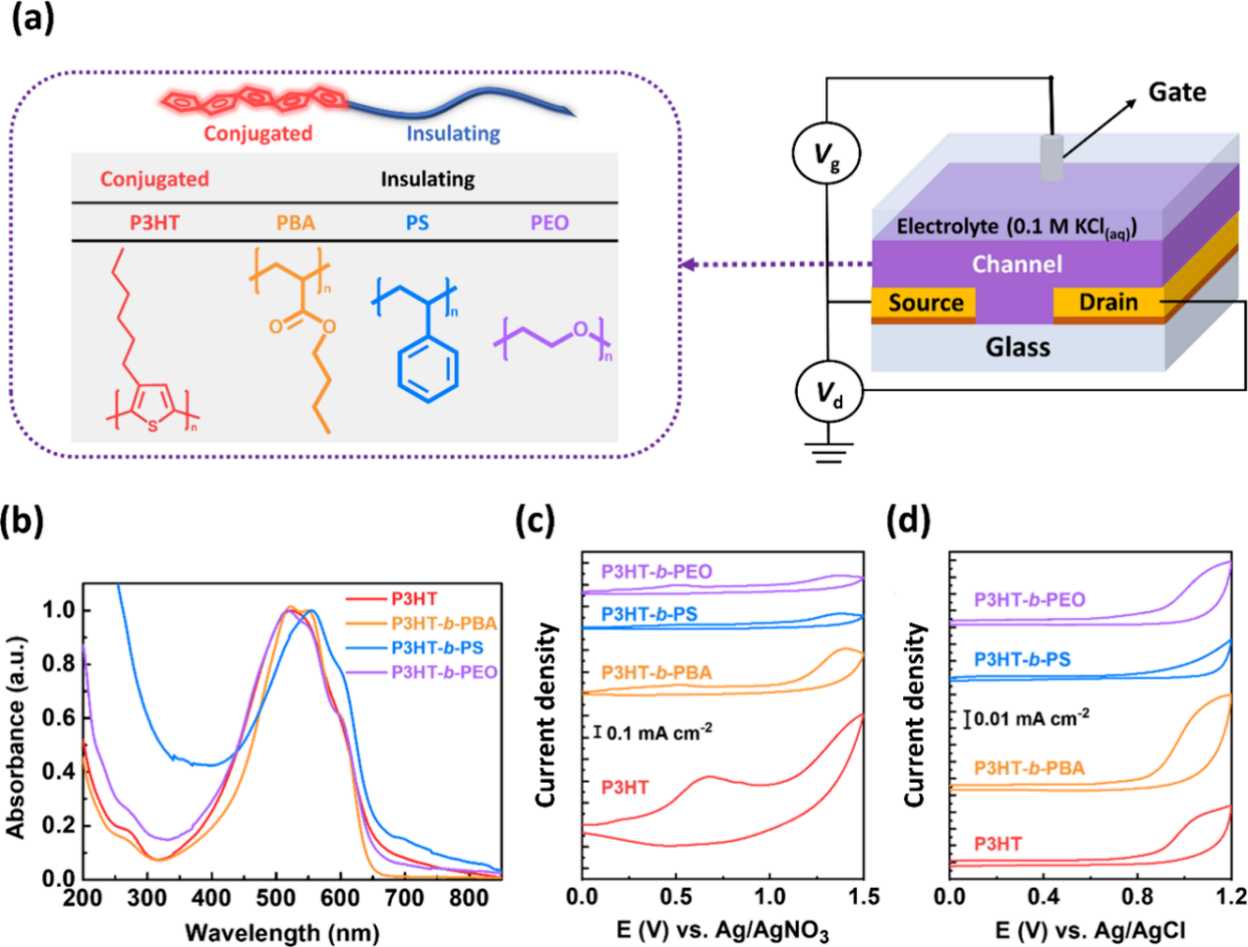
(a) Chemical structure of P3HT and BCPs and the device architecture of an OECT. (b) Optical absorption spectrum of P3HT and BCPs thin films. Cyclic voltammetry of P3HT and BCPs thin films in an electrolyte of (c) 0.1 M tetrabutylammonium perchlorate (TBAP) in acetonitrile or (d) 0.1 M KCl_(aq)_ with a normalized current density.

To investigate the impact of the BCP structure on the performance of the OECT, three types of P3HT-based BCPs were used with varying coils: PBA, PS, and PEO were synthesized. According to the contact angles in Figure S1 and the glass transition points of the insulating blocks,^32,35^ three BCPs are categorized by their different hydrophilicity and rigidity: P3HT-*b*-PBA as a hydrophobic (106.3°) and soft (*T*_g_ = −53 °C) BCP; P3HT-*b*-PS as a hydrophobic (106.0°) and rigid (*T*_g_ = 89 °C) BCP; and P3HT-*b*-PEO as a hydrophilic (69.0°) and soft (*T*_g_ = −50 °C) BCP. The analysis included optical, electrochemical, morphological, and crystallographic properties. In Figure 1a, the chemical structures of the BCPs and the architecture of the top-gate/bottom-contact OECT device are depicted. OECT measurements utilized 0.1 M aqueous potassium chloride (KCl_(aq)_) as the electrolyte. Figure 1b presents the optical characteristics of the BCPs and P3HT homopolymer films, showcasing the absorption onset of the UV–vis spectra and summarizing the optical bandgaps in Table S1. Two distinct absorption bands appear at 230–320 nm and 450–700 nm. The former corresponds to the absorption of insulating blocks, while the latter exhibits absorption peaks at around 600 nm and 520–550 nm, which represent the 0–0 and the 0–1 absorption peaks, respectively.^38^ All polymers display similar absorption onsets at around 650 nm, indicating uniform optical bandgaps of approximately 1.90 eV. This implies that the insulating block in a BCP does not significantly affect its bandgap value, which is dominated by the conjugated block of P3HT. The electrochemical properties and energy levels of the BCPs were explored using cyclic voltammetry (CV), as shown in Figure 1c. The HOMO and LUMO levels were calculated from the CV onset potential in a nonaqueous electrolyte, with values summarized in Table S1. All polymers show similar energy levels, so the conjugated block dominates the energy level of a BCP. Remarkably, the HOMO levels of these BCPs closely match the work function of gold (approximately −5.1 eV), facilitating efficient charge transport from the BCP to the source/drain electrode.^39^ An electrolyte comprising TBAP/acetonitrile is generally identified as a compatible organic electrolyte for characterizing conjugated polymer films. Therefore, this electrolyte was applied to observe the reversible redox properties of the BCPs. The oxidative onset gap between it and an aqueous electrolyte indicates the barrier of anion doping in an aqueous OECT. Accordingly, CVs in aqueous media were measured and presented in Figure 1d to assess the electrochemical behavior of these BCPs in aqueous electrolytes. The onset potentials in aqueous electrolytes shift to higher values due to the energetic barrier at the polymer-electrolyte interface. Table S1 details the difference between the onset potentials in aqueous (*E*_onset,aq_) and organic (*E*_onset,org_) electrolytes. The (*E*_onset,aq_–*E*_onset,org_) values for P3HT, P3HT*-b-*PBA, P3HT*-b-*PS, and P3HT*-b-*PEO are 0.27, 0.17, 0.32, and 0.23 V, respectively. The soft BCPs, P3HT*-b-*PBA and P3HT-*b*-PEO, present a lower energetic barrier at the interface, possibly due to favorable ion injection in a soft BCP matrix. Furthermore, from the perspective of current density, both P3HT and P3HT-*b*-PBA exhibit superior electrochemical activity in organic phase solutions (good inherent charge transport). However, in aqueous solutions, all polymers’ oxidation currents decrease, indicating an interfacial energy barrier between the polymer and the electrolyte. The soft P3HT-*b*-PEO and P3HT-*b*-PBA can maintain a certain current density level, suggesting that soft BCPs can effectively reduce the interfacial energy barrier. Concerning the doping propensity and barrier of a KPF_6_-based electrolyte to these BCPs, it can be predicted that they will be intermediate between that of a KCl-based electrolyte and that of an organic electrolyte, considering the slightly hydrated PF6^–^ ion compared to that of the Cl^–^ ion.

### Spectro-Electrochemical Characterizations

**Figure 2:**
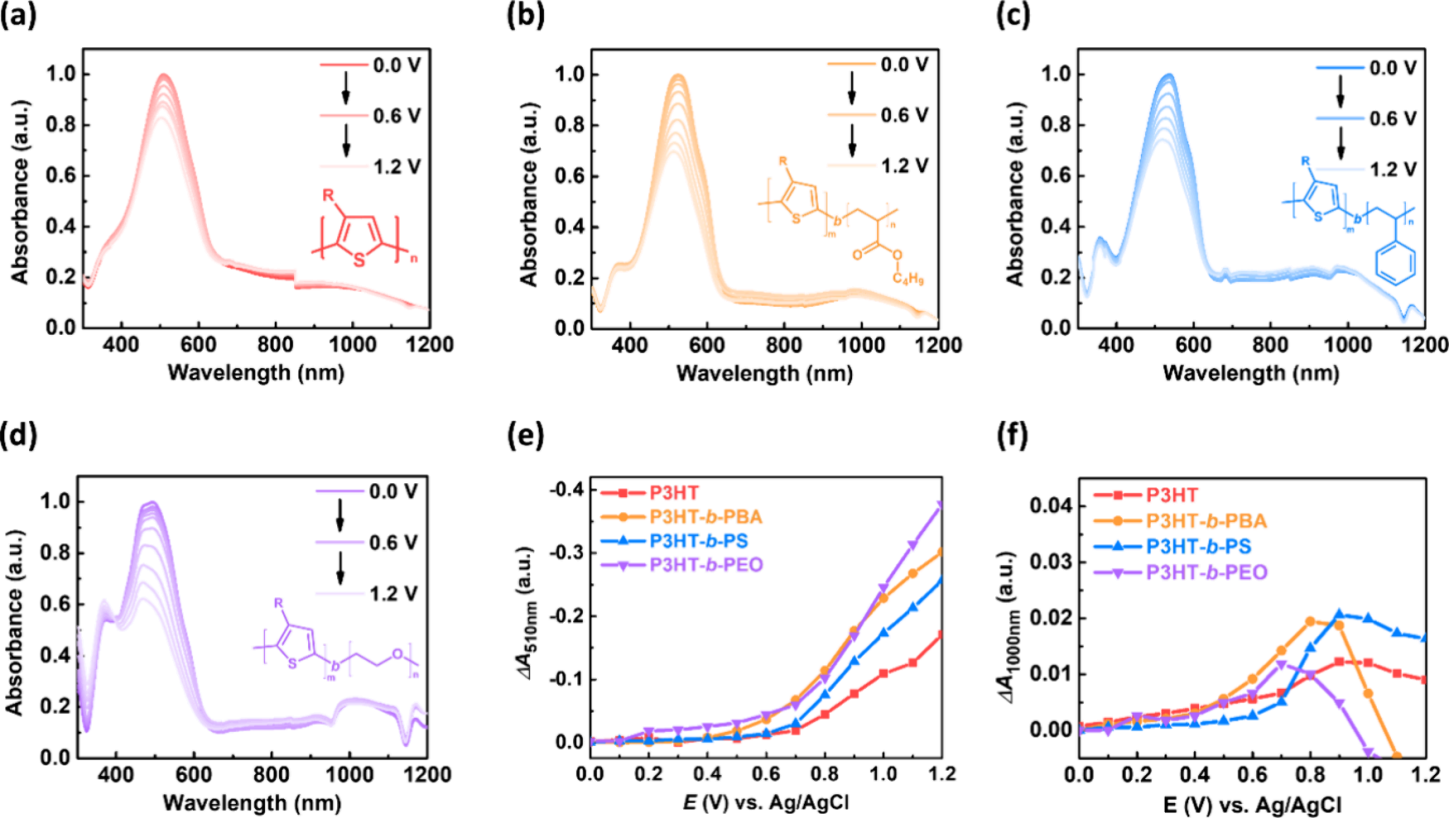
In situ electrochemical optical spectra of (a) P3HT, (b) P3HT-*b*-PBA, (c) P3HT-*b*-PS, and (d) P3HT-*b*-PEO. The extracted changes in optical absorbances at (e) 510 and (f) 1000 nm of the P3HT and BCPs thin films at different potentials.

Following the individual analyses of their optical and electrochemical properties, in situ spectro-electrochemical measurements were conducted to comprehensively investigate the doping behavior of the BCPs under various electrochemical potentials. Figure 2a–d displays the in situ electrochemical optical spectra of the BCPs. The absorption bands at 450–700 nm, representing intramolecular charge transfer (ICT), as known as 0–0 and 0–1 absorption mentioned above, and the absorption band around 1000 nm, indicative of polaron/bipolaron formation, were identified.^40^ With increasing applied potential, the ICT absorption gradually decreased, while the polaron/bipolaron absorption started to increase, aligning with the doping mechanism of the CP.^41^ To quantify their doping behavior, the relative changes in the ICT absorption peak (Δ*A*_510 nm_) and polaron/bipolaron absorption peak (Δ*A*_1000 nm_) were compared, as summarized in Figure 2e–f. At 0.8 V vs Ag/AgCl, corresponding to the onset potential of their CVs in KCl_(aq)_, the ICT absorption of all polymers significantly decreased. P3HT*-b-*PBA and P3HT*-b-*PEO exhibited a more pronounced reduction in ICT absorption and a more significant increase in polaron absorption at a lower electrochemical potential than the P3HT homopolymer. Conversely, P3HT*-b-*PS showed a decreased ICT absorption and increased polaron absorption at a slightly higher potential than the P3HT homopolymer, attributed to its lower doping onset than P3HT*-b-*PBA and P3HT*-b-*PEO. This disparity might lead to more carriers and higher conductivity in the device application. Interestingly, all four CPs displayed decreased polaron and ICT absorption at high potential, suggesting the underlying irreversible oxidation. Consequently, it is advisable to operate within potentials lower than 1 V. The results underscore the connection between doping mechanisms and oxidative onset potential in p-type CPs. A BCP with a lower oxidation onset facilitates a more straightforward doping mechanism, resulting in higher conductivity.

### OECT Device Characteristics in the KCl-Based Electrolyte

**Figure 3:**
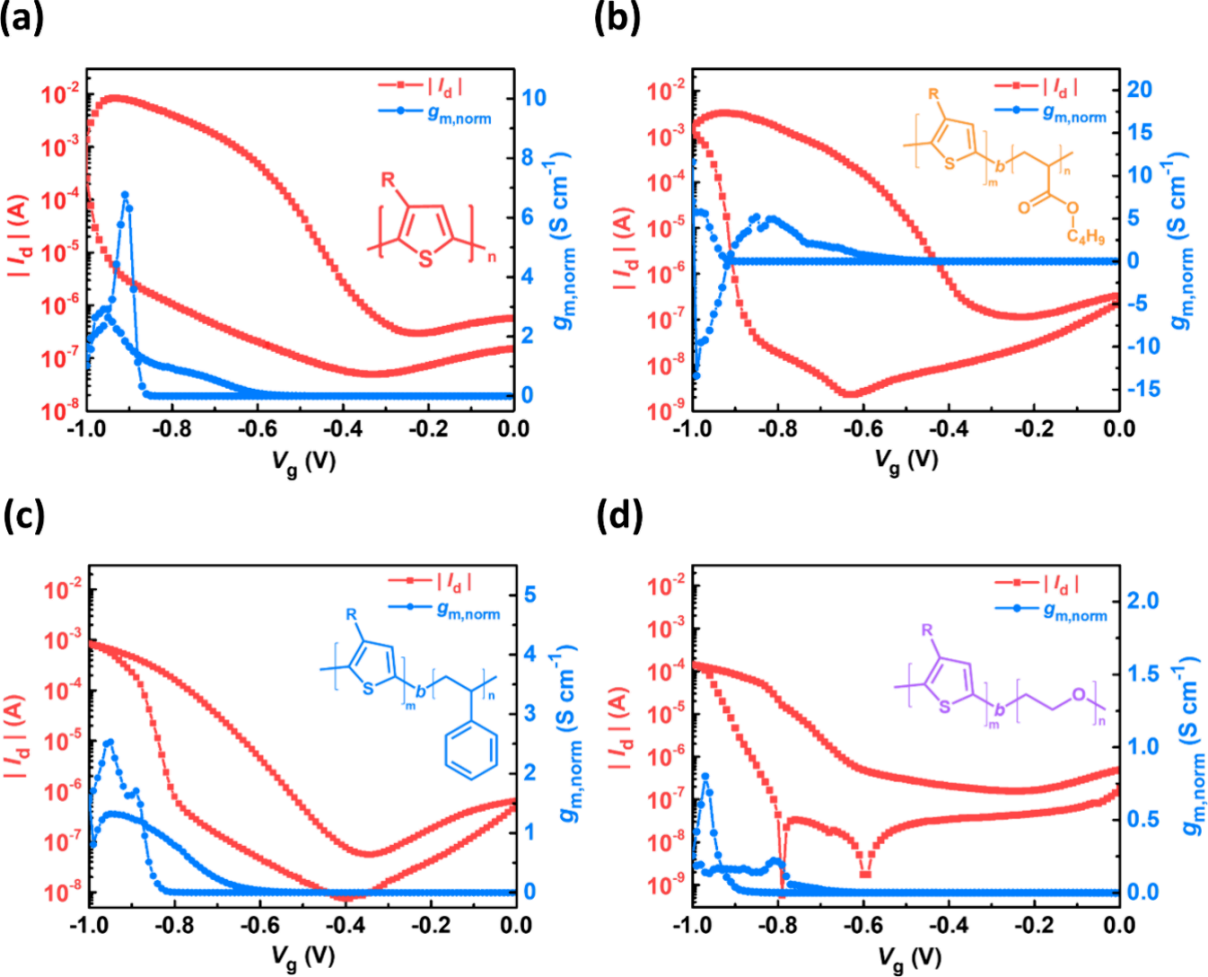
Transfer characteristics of (a) P3HT, (b) P3HT-*b*-PBA, (c) P3HT-*b*-PS, and (d) P3HT-*b*-PEO-based OECT devices in 0.1 M KCl_(aq)_ with *V*_d_ = −0.5 V and forward *V*_g_ swept from 0 to −1.0 V.

**Figure 4:**
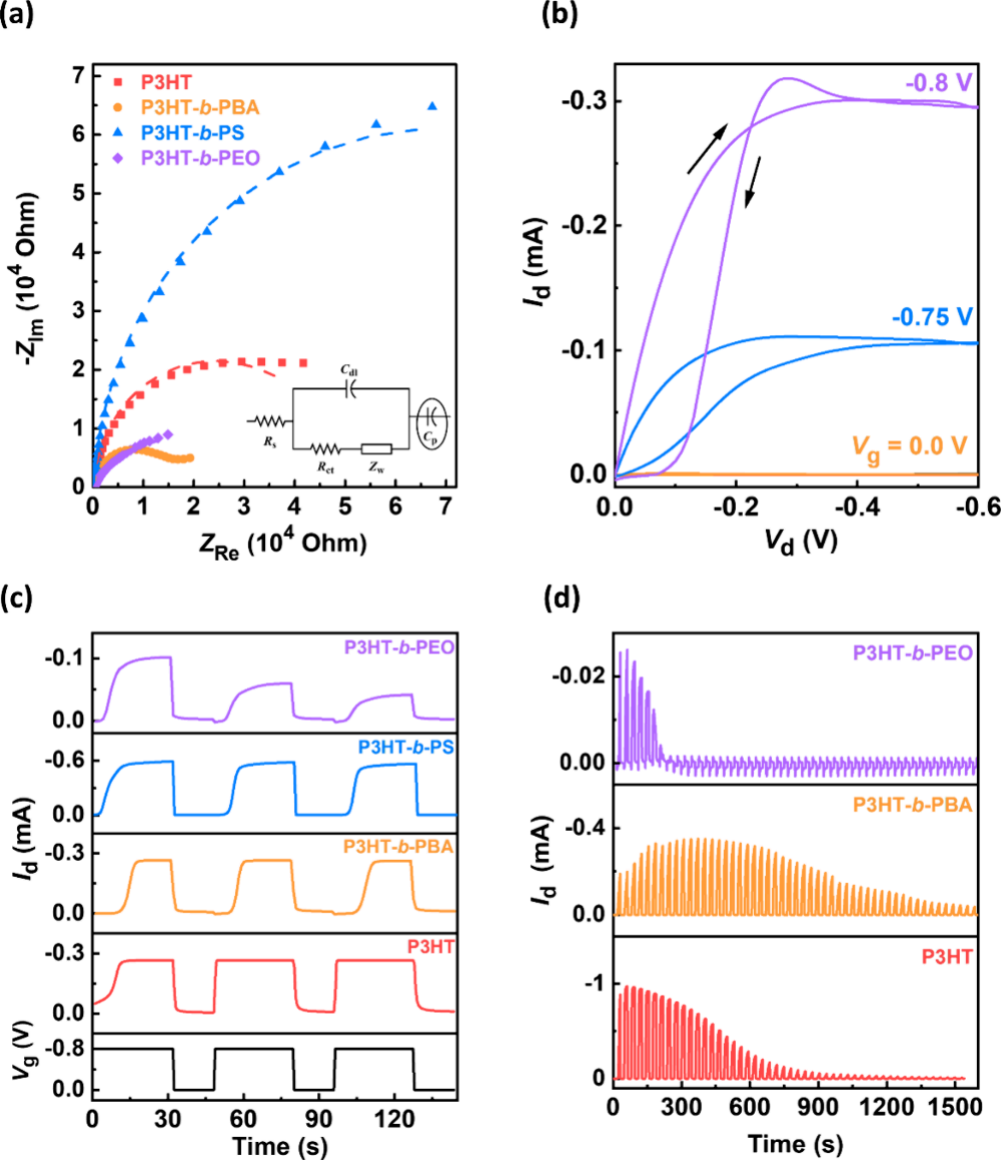
(a) Nyquist plots of the P3HT and BCPs thin films: the dashed line standing for the fitting curve based on the inset equivalent circuit. (b) Output curve of the P3HT-*b*-PBA-based OECT device. (c) Transient characteristics of the BCPs at *V*_d_ = −0.5 V. (d) Stability test of P3HT, P3HT-*b*-PBA, and P3HT-*b*-PEO in 50 cycles at *V*_d_ = −0.1 V and *V*_g_ switched between 0 and −0.9 V.

**Figure 5:**
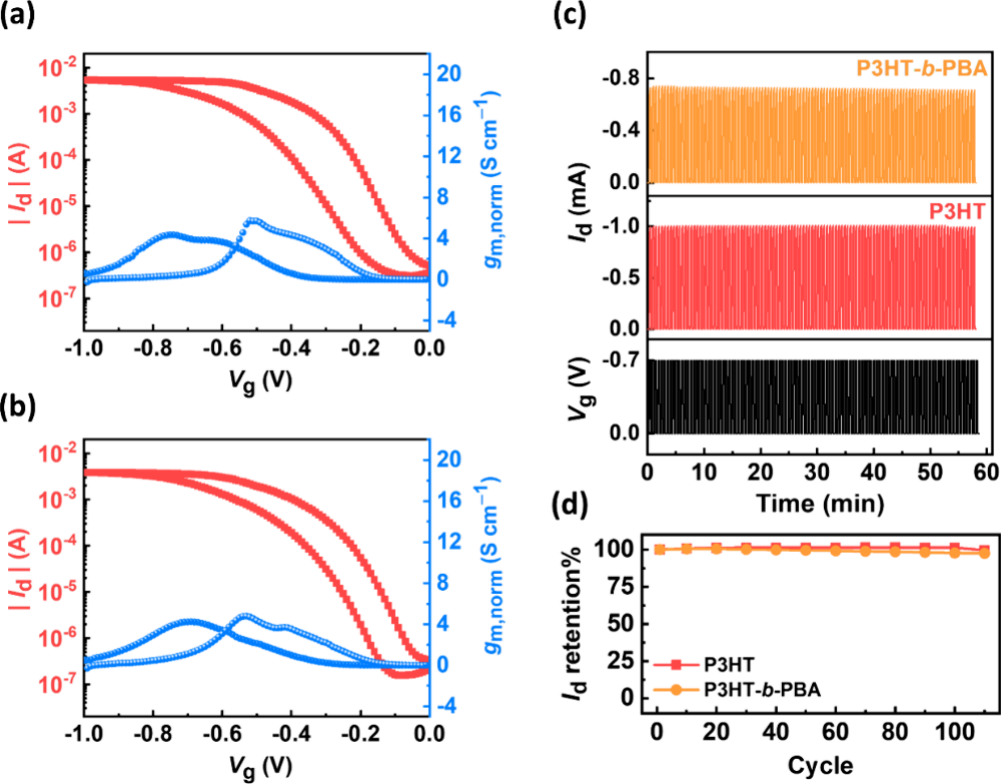
Transfer characteristics of (a) P3HT and (b) P3HT-*b*-PBA-based OECT devices in 0.1 M KPF_6(aq)_ with *V*_d_ = −0.5 V and forward *V*_g_ swept from 0 to −1.0 V. (c) Stability test of P3HT and P3HT-*b*-PBA in 110 cycles in 0.1 M KPF_6(aq)_ at *V*_d_ = −0.1 V and *V*_g_ = −0.7 V. (d) Comparison of the *I*_d_ retention of P3HT and P3HT-*b*-PBA for 110 cycles.

To assess the mixed ionic and electronic transport properties of BCPs in comparison to a homopolymer, OECT characterizations were conducted with P3HT and BCPs as the channel layer, as illustrated in Figure 1a. Gold served as the source and drain electrodes, interdigitated with a channel width (*W*) of 9000 μm (nine parallel channels having a width of 1000 μm) and a length (*L*) of 25 μm. The gate electrode consisted of a Ag/AgCl pellet, and the electrolytes used were 0.1 M KCl_(aq)_ (Figures 3 and 4 and Table 1) or 0.1 M KPF_6(aq)_ (Figure 5 and Table 2) contained in a polydimethylsiloxane (PDMS) well. The film thickness (*d*) of the BCPs, measured by using a surface profilometer, is shown in Table 1. Figure 3 illustrates the transfer curves of the BCPs. All BCPs exhibited typical p-type enhancement-mode characteristics with an increasing drain current (*I*_d_) as the applied gate voltage (*V*_g_) became more negative. Within the same range of *V*_g_, P3HT*-b-*PBA demonstrated the highest *I*_d_, while P3HT*-b-*PEO exhibited the lowest. In addition, 150 °C annealed P3HT*-b-*PBA for the OECT was conducted to examine its performance. The transfer curve shown in Figure S2a is similar to that of the nonannealed one, indicating that the P3HT*-b-*PBA-based OECT device does not need postannealing engineering. According to the literature, annealed CPs may not be feasible for OECT because the crystalline domains will be disrupted by doped ion/water molecules (hydration).^26^ Therefore, all of the OECTs were measured with their nonannealed state. Moreover, to ensure that oxygen reduction does not affect the OECT devices, a degassed electrolyte was utilized in the P3HT*-b-*PBA-based OECT (Figure S2b). The results suggest that the possible oxygen reduction does not influence the device’s performance. It is possibly because the oxygen concentration is low in the electrolyte, and the potential reaction occurs at the gate electrode side. It mitigates the damage by the oxygen. Then, transfer curves at varying fixed *V*_d_ were measured to determine the *V*_d_ when measuring transfer curves (Figure S2c). The transferred *I*_d_ reaches its highest value at *V*_d_ = −0.5 V, possibly because of the strong lateral driving force that makes the doping process difficult. To warrant the OECT operation in the saturation region, the *V*_d_ is set at–0.5 V. Accordingly, Table 1 summarizes the device parameters for the polymers. The threshold voltages (*V*_th_) for P3HT, P3HT*-b-*PBA, P3HT*-b-*PS, and P3HT*-b-*PEO are–0.93,–0.87,–0.82, and–0.81 V, respectively, extrapolated from the linear regime of the *I*_d_^1/2^ versus *V*_g_ plot, as shown in Figure S3. All BCPs displayed values higher than their onset potentials calculated from CV in KCl_(aq)_. There are two possible reasons: the first is the need for carrier accumulation to induce *I*_d_, as hole-limited transport when insufficient carrier generation,^17^ and the second is a significant energy barrier at the hydrophobic polymer-electrolyte interface as mentioned above. Notably, all BCPs exhibited lower *V*_th_ values than the homopolymer, with P3HT*-b-*PEO having the lowest *V*_th_, attributed to a low energetic barrier at the polymer/electrolyte interface and easy counterion injection during the doping process.

**Table 1:** Summary of the Device Performance Metrics of P3HT and BCPs-Based OECTs in 0.1 M KCl_(aq)_

polymer	*d* (nm)^a^	*g*_m,norm_ (S cm^–1^)^b^	*V*_th_ (V)^c^	*p* (× 10^21^ cm^–3^)^d^	μC* (F s^–1^ cm^–1^ V^–1^)^e^	C* (F cm^–3^)^f^	μ (cm^2^ s^–1^ V^–1^)^g^	*t*_r_ (s)^h^	*t*_f_ (s)^h^
P3HT	93.0 ± 2.82	7.55 ± 4.50	–0.93 ± 0.01	1.24 ± 0.11	58.2 ± 11.4	110 ± 22.2	0.529	1.50 ± 1.03	0.82 ± 0.17
P3HT-*b*-PBA	69.0 ± 4.73	10.4 ± 1.26	–0.87 ± 0.03	1.33 ± 0.15	170 ± 40.9	328 ± 65.7	0.518	4.09 ± 1.16	0.78 ± 0.13
P3HT-*b*-PS	70.5 ± 2.00	3.52 ± 1.07	–0.82 ± 0.01	2.78 ± 0.25	38.3 ± 8.26	454 ± 86.6	0.084	3.18 ± 0.94	0.82 ± 0.01
P3HT-*b*-PEO	66.2 ± 8.68	1.16 ± 0.32	–0.81 ± 0.01	1.70 ± 0.25	4.83 ± 1.13	71.0 ± 5.98	0.068	5.00 ± 0.22	2.88 ± 0.11

a Film thickness measured by a surface profilometer.

b Transconductance normalized by the channel dimension (*Wd*/*L*).

c Threshold voltage extracted from the *x*-intercept of *I*_d_^1/2^ as a function of *V*_g_.

d Carrier density calculated by the integrated area of the *I*_g_ versus *V*_g_ curve and normalized by the dimension.

e OECT figure of merit: product of mobility and volumetric capacitance extracted from the slope of *g*_m_ as a function of (*Wd*/*L*)(*V*_th_–*V*_g_).

f Volumetric capacitance obtained from EIS measurements.

g Mobility calculated by using the C* values.

h Rise time and fall time obtained by the transient curve fitted by using an exponential decay function.

**Table 2:** Summary of the Device Performance Metrics of P3HT and P3HT-*b*-PBA-Based OECTs in 0.1 M KPF_6(aq)_

polymer	*g*_m,norm_ (S cm^–1^)	*V*_th_ (V)	*p* (× 10^21^ cm^–3^)	μC* (F s^–1^ cm^–1^ V^–1^)	*t*_r_ (s)	*t*_f_ (s)
P3HT	4.04 ± 1.94	–0.31 ± 0.03	1.46 ± 0.56	8.62 ± 4.93	1.66 ± 0.46	0.43 ± 0.01
P3HT-*b*-PBA	3.73 ± 0.75	–0.31 ± 0.01	1.70 ± 0.23	9.18 ± 1.38	1.94 ± 0.20	0.31 ± 0.00

The normalized transconductance (*g*_m,norm_), a key parameter for evaluating OECT transconductance efficiency, was determined by differentiating *I*_d_ with respect to *V*_g_ and normalizing it by the channel dimension (*Wd*/*L*). The *g*_m,norm_ values for P3HT, P3HT*-b-*PBA, P3HT*-b-*PS, and P3HT*-b-*PEO are 7.55, 10.4, 3.52, and 1.16 S cm^–1^, respectively. P3HT*-b-*PBA exhibited the highest *g*_m,norm_ value, indicating superior amplification within the same voltage regime. Subsequently, the product of charge-carrier mobility (μ) and volumetric capacitance (C*), denoted as μC*, serves as a benchmark for evaluating the mixed ionic–electronic transport properties of the BCPs, as presented in Table 1. The μC* values were derived from the slope of the linear fit to the corresponding plot of *g*_m_ versus *WdL*^–1^(*V*_th_–*V*_g_) (Figure S4), and the fitting ranges from *V*_th_ to its highest slope of *g*_m_ to confirm the average condition. The μC* values for P3HT, P3HT*-b-*PBA, P3HT*-b-*PS, and P3HT*-b-*PEO are 58.2, 170, 38.3, and 4.83 F s^–1^ cm^–1^ V^–1^, respectively. To estimate the quantity of charge carriers generated when applying gate bias, the curves of the gate current (*I*_g_) versus *V*_g_ were integrated (Figure S5) and converted into carrier density (*p*).^19^ The *p* values for P3HT, P3HT*-b-*PBA, P3HT*-b-*PS, and P3HT*-b-*PEO are 1.24 × 10^21^, 1.33 × 10^21^, 2.78 × 10^21^, and 1.70 × 10^21^ cm^–3^, respectively. P3HT-*b*-PS exhibited the highest *p*, undergoing the most oxidation and expectedly generating the most charge carriers in a unit volume. From their gate currents, Figure S6 shows the full-scan results of the gate current. P3HT and P3HT*-b-*PBA show early onset oxidation in slow-scan mode; therefore, peaks appear at around–0.9 and–1.0 V. The reverse scan curves show that all CPs undergo irreversible oxidation to some extent as there is no or low reduction current. This is inevitable because of its large interfacial barrier to be doped/dedoped, as described in the previous part of electrochemical behaviors in aqueous KCl. These OECT characterizations indicate that P3HT*-b-*PBA exhibited the best μC*, and it is better than other reported P3HTs for the active layer in OECTs (as shown in Table S2). With regard to P3HT*-b-*PS, it displayed similar results to those of the P3HT homopolymer. Conversely, P3HT*-b-*PEO shows the least favorable mixed ionic and electronic transport properties.

Electrochemical impedance spectroscopy (EIS) was conducted to evaluate the capacitive behaviors to obtain C* to deconvolute the μC* product. Figure 4a presents the Nyquist plot of the P3HT and BCPs films. The EIS was measured at 1.0 V and fitted by the equivalent circuit presented in Figure 4a. The fitting results show that the volumetric capacitances of P3HT, P3HT*-b-*PBA, P3HT*-b-*PS, and P3HT*-b-*PEO are, respectively, 110, 328, 454, and 71.0 F cm^–3^ (Table 1). This result is related to their carrier density, as shown above. Because P3HT-*b*-PS generated the highest quantity of charge carrier, it showed the highest C*, except for that of P3HT-*b*-PEO. The possible reasons for this will be discussed below. Then, the mobility was extracted from the μC* product, as shown in Table 1. The μ values of P3HT, P3HT*-b-*PBA, P3HT*-b-*PS, and P3HT*-b-*PEO are 0.529, 0.518, 0.084, and 0.068 cm^2^ s^–1^ V^–1^, respectively. P3HT*-b-*PBA exhibited similar μ values with the P3HT homopolymer, indicating that the soft/hydrophobic PBA block did not interfere with the carrier transport. In contrast, the soft/hydrophilic PEO block significantly deteriorated the carrier transport. This disparity is possibly attributed to the disordered and swelled structure by electrolyte and ion doping. Regarding the P3HT*-b-*PS, the rigid/hydrophobic may hinder the solid-state stacking of the P3HT block, thereby confining the carrier transport.

To avoid confusion and overestimation of mobility value obtained by varying methods in OECT,^42^ the mobility values were further determined by the transient technique for each OECT.^43^Figure S7 presents their transient characteristics by applying different gate currents, and Figure S8 displays the fitting result of the reciprocal transit time (−1/τ_e_). Then, mobility is calculated by .^36,37^ The calculated mobility for P3HT, P3HT*-b-*PBA, P3HT*-b-*PS, and P3HT*-b-*PEO is 2.79 × 10^–3^, 6.13 × 10^–3^, 5.53 × 10^–3^, and 1.04 × 10^–4^ cm^2^ s^–1^ V^–1^, respectively. The mobility values are strictly lower by 1 to 2 orders than those calculated by linear fitting of *g*_m_ vs *WdL*^–1^(*V*_th_–*V*_g_). There are two possible reasons: high *V*_th_ value of the OECT device or nonideal channel and contact resistance. High *V*_th_ due to hole-limited carrier transport^17^ and a large polymer-electrolyte energy barrier will make the transconductance overestimated with too dramatically increased carrier transport. Remarkably, P3HT*-b-*PS presents the same order of mobility as P3HT and P3HT*-b-*PBA, indicating that the slope-fitting method underestimates the mobility of P3HT*-b-*PS because it has superior charge storage capability and lower *V*_th_, and the calculated mobility by slope-fitting is much lower. However, P3HT*-b-*PBA still exhibits the best OECT characteristics. Also, the carrier density method is applied to derive the mobility: ,^18,37^ where the drain current (*I*_d_) is averaged from *V*_th_ to–1.0 V from the transfer curve. The average mobility derived from this method for P3HT, P3HT*-b-*PBA, P3HT*-b-*PS, and P3HT*-b-*PEO is 4.17 × 10^–4^, 2.84 × 10^–3^, 7.28 × 10^–4^, and 8.49 × 10^–5^ cm^2^ s^–1^ V^–1^, respectively. All mobility and μC* values determined by each method are summarized in Table S3. These mobilities calculated by the current density are close to those by the transient method and follow the same trend, but the μC* values differ with these methods.

The output characteristics of the BCPs are presented in Figures 4b and S9, indicating that all polymers initiate drain currents at *V*_g_ = −0.75 V. In addition, the drain currents of the BCPs reached the saturation regime when *V*_d_ was approximately at −0.5 V. Their saturated *I*_d_s are approximately −0.225, −0.300, −0.550, and −0.075 mA for P3HT, P3HT*-b-*PBA, P3HT*-b-*PS, and P3HT*-b-*PEO, respectively. To explore the response time of these BCPs, the transient curves applying switched *V*_g_, 0 and −0.8 V with a switch length of 15 and 30 s, were carried out and are presented in Figure 4c. The rise time (*t*_r_) and fall time (*t*_f_) were estimated by exponential fitting (as shown in Figure S9) and are displayed in Table 1. The saturated *I*_d_s are similar to those in output curves. The *t*_r_s of P3HT, P3HT*-b-*PBA, P3HT*-b-*PS, and P3HT*-b-*PEO are 1.50, 4.09, 3.18, and 5.00 s and the *t*_f_s are 0.82, 0.78, 0.82, and 2.88 s, respectively. The doping kinetics of BCPs prove to be slower than those of the homopolymer, with soft BCPs exhibiting a slower response compared with the rigid one. This is related to their crystallinity, which will be discussed following. The higher crystallinity results in faster hole transport and then shows a faster response. Remarkably, P3HT-*b*-PEO displayed a decayed drain current in the transient curve, suggesting poor stability and a low μC*. Device stability was assessed through continuous *V*_g_ switching between 0 and −0.9 V, with a switching duration of 15 s. *V*_d_ was set at −0.1 V to minimize the lateral driving force, as shown in Figure 4d. Figure S11 illustrates the *I*_d_ retention of P3HT, P3HT-*b*-PBA, and P3HT-*b*-PEO across each cycle compared with their first cycle. After 50 consecutive switching cycles, P3HT-*b*-PBA exhibits a higher retention of 21% than P3HT with 1%, and P3HT-*b*-PEO already failed without response. It should be noted that the drain currents of P3HT-*b*-PBA in 10 cycles experience an increase, possibly due to its relatively slow doping kinetics in KCl_(aq)_. However, the decrease in retention of P3HT-*b*-PBA is still slower than that of P3HT. In addition, the retention of P3HT-*b*-PEO significantly decreased after just 10 cycles. These results indicate that P3HT-*b*-PBA has better stability, while P3HT-*b*-PEO demonstrates poor stability. Moreover, multiple cycles of transfer curves were measured to evaluate their device stability, and the results are displayed in Figure S12. From these curves, P3HT exhibits maintenance of its drain current until its 10th cycle, while P3HT-*b*-PBA and P3HT-*b*-PEO maintain drain currents until its 14th cycle and sixth cycle, respectively. This also indicates that P3HT-*b*-PBA has the best stability among these CPs, and P3HT-*b*-PEO is the worst.

### OECT Device Characteristics in the KPF_6_-Based Electrolyte

The BCPs’ device properties were further characterized using KPF_6_ as the electrolyte because the hydration of Cl^–^ ions will cause a low doping efficiency. Polarizable and hydrophobic PF_6_^–^ can avoid hydration and, hence, high doping efficiency for a hydrophobic CP.^26^Figure 5a,b shows the transfer curves of OECTs based on P3HT and P3HT-*b*-PBA, operated in 0.1 M aqueous KPF_6_. Table 2 displays their device performance parameters in KPF_6(aq)_. As can be seen in Table S2, P3HT generally exhibited high *V*_th_ values in KCl-based aqueous electrolytes. This phenomenon is attributed to P3HT’s strong hydrophobicity and interdigitated lamellar structures, thereby retarding the doping kinetics of Cl^–^ ions. Compared to their operation in aqueous KCl, both P3HT and P3HT-*b*-PBA exhibit lower *V*_th_ values with KPF_6(aq)_ as the electrolyte, suggesting that the Cl^–^ ion has poor doping efficiency and speed with hydrophobic BCPs. P3HT-*b*-PBA demonstrates slightly better μC* values than P3HT in KPF_6(aq)_ because of the design of soft and hydrophobic blocks. Moreover, as shown in Figure 5c,d, both P3HT and P3HT-*b*-PBA exhibit good stability in KPF_6(aq)_ over 55 min and 110 cycles, indicating the superior doping behavior of PF_6_^–^ compared to Cl^–^. The significantly different performance of P3HT and P3HT-*b*-PBA in these two electrolytes is due to their hydrophobic nature, thereby retarding the doping kinetics of the highly hydrated Cl^–^ ions compared with the slightly hydrated PF_6_^–^ ions. The improved performance of P3HT-*b*-PBA in KPF_6_-based electrolytes is attributed to its soft and hydrophobic backbone, which contributes to the stability of its backbone structure, ensuring enhanced capacitance during the OECT operation; therefore, it warrants more stable and faster device operations under low voltages in KPF_6_-based electrolytes than that in KCl-based electrolytes. To further improve BCPs’ performance in KCl_(aq)_, introducing hydrophilic side chains to these BCPs could be a feasible strategy to address this issue. Nevertheless, P3HT-*b*-PBA still exhibits the best performance and stability in KCl_(aq)_, which can emphasize the advantage of P3HT-*b*-PBA due to its better morphology and doping behavior.

### Morphological and Crystallographic Properties of the KCl-Based Electrolyte-Swelled Films

**Figure 6:**
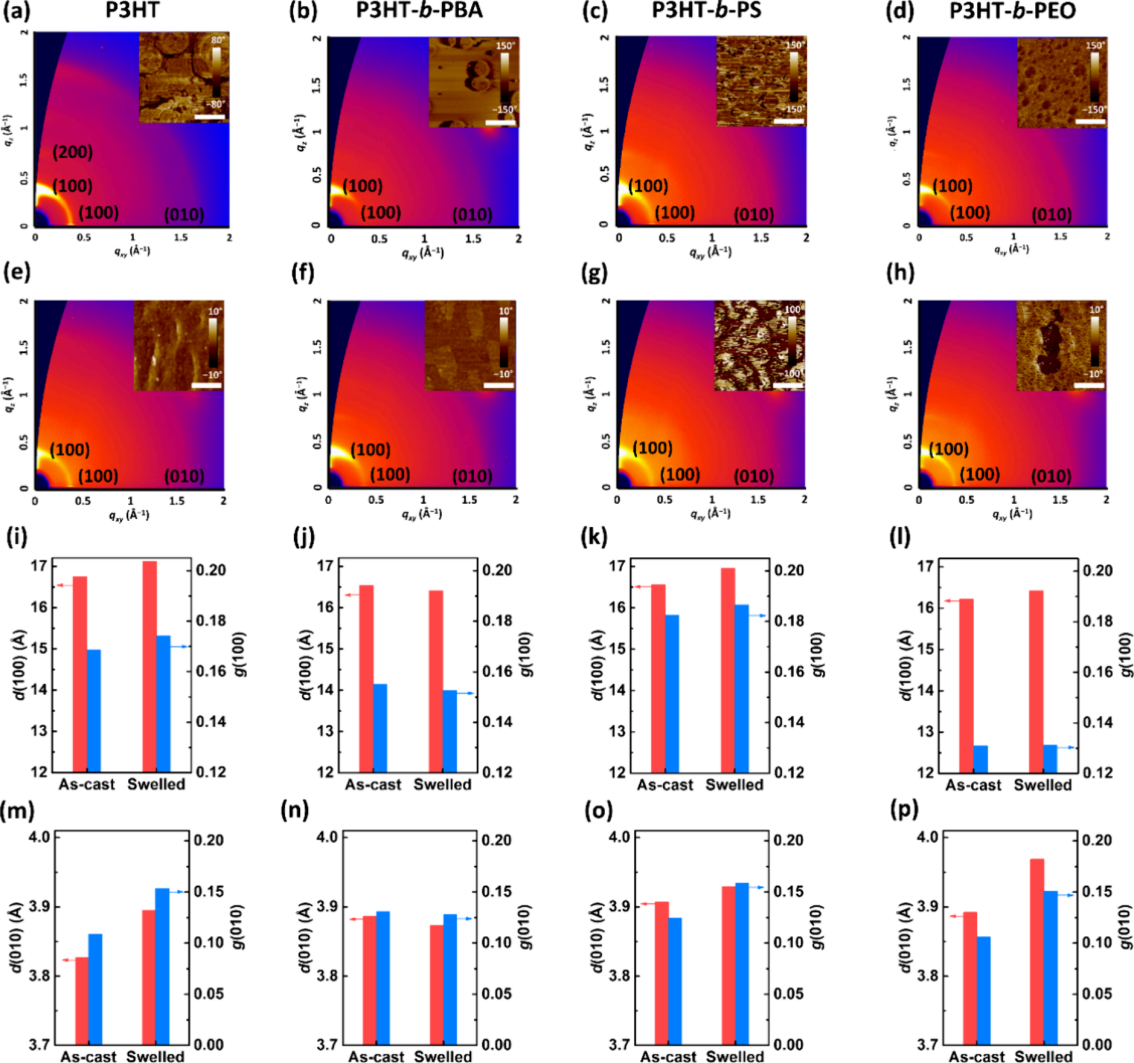
2D GIXD patterns of the (a–d) as-cast films and (e–h) electrolyte-swelled films of (a,e) P3HT, (b,f) P3HT-*b*-PBA, (c,g) P3HT-*b*-PS, and (d,h) P3HT-*b*-PEO. The upper-right insets are the AFM phase images with a scale bar of 2 μm. Crystallographic parameters including the *d*-spacing and paracrystalline disorder of the (i–l) out-of-plane (100) and (m–p) in-plane (010) for (i,m) P3HT, (j,n) P3HT-*b*-PBA, (k,o) P3HT-*b*-PS, and (l,p) P3HT-*b*-PEO. 0.1 M KCl_(aq)_ (0.1 M) was used as the electrolyte to swell the polymer films.

To figure out how the BCP structures influence the OECT performance, GIXD and AFM of these BCPs were carried out, and Figure 6a–h presents the 2D GIXD patterns and AFM images for the as-cast (a–d) and the swelled (e–h) thin films. From the AFM images, the BCPs showed porous structures, with increased pore sizes observed after the swelling process, indicating film expansion upon immersion in the electrolyte. Notably, the P3HT-*b*-PEO film was peeled off from the substrate after swelling, resulting in the disconnection of each polymer chain and, hence, poor charge transport properties. This is possibly due to its low contact angle, and some PEO chains dissolve into the electrolyte. Thus, hydrophilic insulating blocks are unsuitable for BCP design in OECT applications. For the GIXD analysis, Figure S13 displays the 1D GIXD profiles in the out-of-plane (OOP) and the in-plane (IP) directions and the geometrically corrected pole figures. All BCPs showed a predominate edge-on orientation, and the edge-on and face-on populations of P3HT-*b*-PS and P3HT-*b*-PEO decreased after swelling. In contrast, P3HT-*b*-PBA showed an increased edge-on population, which enhanced its carrier mobility. The crystallographic parameters, including *d*-spacing (*d*) and paracrystalline disorder (*g*), are summarized in Table 3. The crystallite coherence length (*L*_c_) and relative degree of crystallinity (rDOC) are summarized in Table S4. Figure 6i–l displays the comparison of *d* and *g* of the OOP (100) (lamellar stacking) diffractions of P3HT and BCPs, and Figure S14a–d displays the comparison of *L*_c_ of the OOP (100) diffractions of P3HT and BCPs. All of the as-cast films showed similar *d*-spacings. However, the *d*-spacings of the swelled films increased, except for that of P3HT-*b*-PBA; and P3HT-*b*-PEO also presented a slightly increased *d*-spacing. Next, the Scherrer equation estimated the coherence length based on the OOP (100) direction,^44^ as shown in Figure S13a,d. The resulting *L*_c_s of P3HT, P3HT*-b-*PBA, P3HT*-b-*PS, and P3HT*-b-*PEO at their as-cast state are 84.3, 98.6, 71.3, and 135.4 Å, and the *L*_c_ values of the BCPs at their swelled state are 80.8, 100.9, 69.7, and 136.4 Å, respectively. The soft BCPs undergo an increased crystallite size after swelling in the electrolyte. The paracrystalline disorder is used to evaluate their packing order.^45^ The values are 0.169, 0.155, 0.182, and 0.131 for the as-cast films and 0.174, 0.153, 0.187, and 0.131 for the swelled films of P3HT, P3HT*-b-*PBA, P3HT*-b-*PS, and P3HT*-b-*PEO, respectively. P3HT*-b-*PEO and P3HT*-b-*PBA showed relatively ordered packing. After swelling, their orders were still well-maintained.

**Table 3:** Summary of the *d*-Spacing (*d*) and Paracrystalline Disorder (*g*) of Lamellar Stacking and π–π Stacking of P3HT and BCPs

polymer	status	*q*_*z*_(100) (Å^–1^)	*d*(100) (Å)^a^	*g*(100)^b^	*q*_*xy*_(010) (Å^–1^)	*d*(010) (Å)^c^	*g*(010)^d^
P3HT	as-cast	0.375	16.7	0.169	1.642	3.83	0.109
	swelled	0.367	17.1	0.174	1.613	3.89	0.153
P3HT-*b*-PBA	as-cast	0.369	16.5	0.155	1.617	3.89	0.131
	swelled	0.379	16.4	0.153	1.622	3.87	0.128
P3HT-*b*-PS	as-cast	0.375	16.6	0.182	1.608	3.91	0.125
	swelled	0.374	17.0	0.187	1.599	3.93	0.159
P3HT-*b*-PEO	as-cast	0.380	16.2	0.131	1.614	3.89	0.106
	swelled	0.383	16.4	0.131	1.583	3.97	0.151

a Lamellar spacing value calculated by *d*(100) = 2π/*q*_*z*_(100).

b Paracrystalline disorder of lamellar stacking calculated by the equation (*g* = (0.5·fwhm·π^–1^·*q*^–1^)^1/2^).

c Lamellar spacing value calculated by *d*(010) = 2π/*q*_*xy*_(010).

d Paracrystalline disorder of π–π stacking.

With regard to the π–π stacking of these polymers, Figures 6m–p and S14e,h present the comparison of crystallographic parameters based on the IP (010) diffractions. The π–π stacking distances of the as-cast films measure 3.83, 3.89, 3.91, and 3.89 Å for P3HT, P3HT-*b*-PBA, P3HT-*b*-PS, and P3HT-*b*-PEO, respectively. Following swelling, their π–π stacking distance experienced slight increases, except for P3HT-*b*-PBA. This phenomenon could be attributed to the potential intercalation of electrolyte molecules. Notably, hydrophilic P3HT-*b*-PEO exhibited the most considerable increased distance, while hydrophobic P3HT-*b*-PBA and P3HT-*b*-PS demonstrated a minimal impact on their *d*-spacings. The *L*_c_s of P3HT, P3HT-*b*-PS, and P3HT-*b*-PEO decreased, suggesting disruption of π–π domains. However, the *L*_c_ of P3HT-*b*-PBA exhibited a slight increase postswelling (from 32.3 to 33.7 Å), resulting in decreased *g* from 0.131 to 0.128 in P3HT-*b*-PBA, while other polymers displayed increased *g* from 0.109 to 0.153 for P3HT, 0.125 to 0.159 for P3HT-*b*-PS, and 0.106 to 0.151 for P3HT-*b*-PEO. Among the P3HT and BCPs, only P3HT-*b*-PBA could maintain the structure of π–π stacking domains, resulting in its relatively high mobility. In contrast, P3HT-*b*-PEO showed a disordered structure due to the hydrophilic coils’ absorption of an excessive number of electrolyte molecules. In addition, rigid P3HT and P3HT-*b*-PS were susceptible to electrolyte swelling and induced a disordered structure.

The rDOC is used to compare their relative amount of repeated packing, assessed by the ratio of the integrated areas of the peaks in the OOP direction in their pole figures (as shown in Figure S13c,f) relative to that of P3HT.^46^Figure S14i–l displays the comparison of rDOC of P3HT and BCPs. The values of P3HT, P3HT*-b-*PBA, P3HT*-b-*PS, and P3HT*-b-*PEO are 1.00, 0.78, 0.82, and 0.58 at the as-cast state and 0.83, 0.88, 0.39, and 0.64 at the swelled state, respectively. For soft P3HT*-b-*PEO and P3HT*-b-*PBA, the relative crystallinity increased after electrolyte swelling, while for rigid P3HT and P3HT*-b-*PS, the relative crystallinity decreased after swelling. This disparity is potentially due to the relatively abundant amorphous regions of soft P3HT*-b-*PEO and P3HT*-b-*PBA to accommodate the intercalated electrolyte. Furthermore, the uniform distribution of electrolyte molecules in the amorphous domains possibly contributed to the improved molecular order.^47^ From the analysis above, we conclude that the soft blocks are favorable for maintaining the pecking order and the relative crystallinity at the swelled state, preventing them from decreasing their mobility.

**Figure 7:**
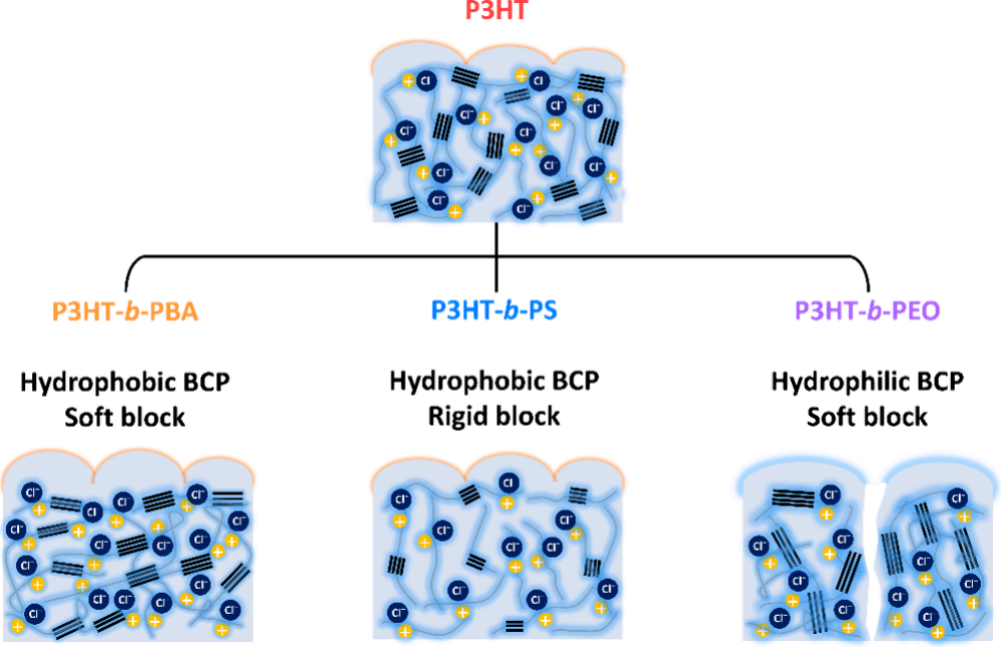
Schematic illustration of the structural influence on OECT performance and stability of the OECT with different insulating blocks related to their structure properties.

In summary of the above analysis with KCl-based electrolytes, Figure 7 illustrates the impacts of structural properties with different insulating blocks on the OECT performance and stability. Based on AFM morphologies, the hydrophilic P3HT-*b*-PEO swelled and delaminated into the electrolyte during device operation, leading to the lowest performance and stability. The GIXD analysis reveals that the rigid P3HT-*b*-PS experiences significant structural disruption upon hydration, leading to relatively low mobility compared with that of P3HT-*b*-PBA. Still, it has better charge storage properties than all other CPs. Conversely, the soft and hydrophobic P3HT-*b*-PBA stabilizes the structure during swelling, showing comparable mobility and improved capacitance compared with the P3HT homopolymer. Based on the discussions, With regard to the structure–morphology–performance relationship of P3HT and BCPs, the stable insulating block warrants its high charge storage performance (capacitance). In contrast, the hydrophilic PEO block significantly deteriorated the carrier transport. This disparity is possibly attributed to the disordered and swelled structure caused by electrolytes and ion doping. Finally, P3HT*-b-*PBA exhibited carrier-transport performance, higher charge storage performance, and device stability comparable to those of the P3HT homopolymer, indicating that the hydrophobic BCP design provides interfacial stabilization without compromising its ionic–electronic conductive properties. In summary, including a hydrophobic block prevents BCP dissolution in the electrolyte, enhancing long-term stability. Moreover, introducing a soft block minimizes the disruption of molecular packing during hydration, preserving the charge transport/storage properties. Concerning the morphological variation engendered by the KPF_6_-based electrolyte to these BCPs, it can be predicted that the swelling and doping of this hydrophobic anion will not significantly degrade P3HT’s crystalline structure in comparison to the KCl-based electrolyte due to the highly hydrated Cl^–^ ions compared with the slightly hydrated PF_6_^–^ ions in the KPF_6_-based electrolyte.

## Conclusions

This study employed P3HT-based BCPs as the active layer in the OECTs to assess their mixed ionic–electronic transport properties and examine the correlation between the BCPs’ hydrophilicity, rigidity, and the OECT performance. The hydrophobicity and crystallographic parameters of the polymers are systematically investigated and correlated to their device performance. Among the BCPs tested, P3HT-*b*-PBA demonstrated the highest μC* at 170 F s^–1^ cm^–1^ V^–1^, surpassing P3HT, P3HT-*b*-PS, and P3HT-*b*-PEO with values of 58.2, 38.3, and 4.83 F s^–1^ cm^–1^ V^–1^, respectively. The excellent performance of P3HT-*b*-PBA warrants high output currents by relying on high voltages in KCl-based electrolytes. Furthermore, P3HT-*b*-PBA exhibited a superior *I*_d_ retention of 21% after 50 cycles compared to the 1% retention observed with the P3HT homopolymer. The exceptional OECT performance of P3HT-*b*-PBA can be attributed to its hydrophobic and soft backbone, promoting charge transport preservation and improved charge storage during swelling. Conversely, a hydrophilic backbone like P3HT-*b*-PEO led to dissolution and poor charge transport in the OECT operation despite favorable swelling properties. The drain current retains only 41% of its first cycle after three cycles for P3HT-*b*-PEO. The hydrophobic P3HT-*b*-PS demonstrated stability in the OECT and achieved the highest capacitance, but its rigid backbone disrupted molecular packing, relatively diminishing charge transport capability compared with P3HT-*b*-PBA. With regard to the performance in the KPF_6_-based aqueous electrolyte, P3HT-*b*-PBA outperforms with a higher μC* of 9.2 F s^–1^ cm^–1^ V^–1^ than that of 8.6 F s^–1^ cm^–1^ V^–1^ observed from P3HT. Notably, both polymers exhibited almost no decay in device performance over 110 ON/OFF switching cycles. The significantly different performance of P3HT-*b*-PBA in these two electrolytes is due to the polymer’s hydrophobicity and interdigitated lamellar structures, thereby retarding the doping kinetics of the highly hydrated Cl^–^ ions compared with the slightly hydrated PF_6_^–^ ions. The excellent performance of P3HT-*b*-PBA in KPF_6_-based electrolytes warrants more stable and faster device operations under low voltages in KPF_6_-based electrolytes than in KCl-based electrolytes. This study marks the first exploration of designing conjugated BCPs for OECT applications and investigating the relationship between BCP structures and enhancements in ionic–electronic conductive properties.

## Supporting Information Available

Summary of optical, electrochemical properties, and crystallographic parameters of the BCP thin films; contact angle images; OECT output characteristics and device fitting parameters of the BCP thin films; and 1D GIXD line-cutting profiles (PDF)
