# Supplementary material for: Interfacial Stabilization of Organic Electrochemical Transistors Conferred Using Polythiophene-Based Conjugated Block Copolymers with a Hydrophobic Coil Design
Source: ACS Appl Mater Interfaces. 2024 Sep 17;16(39):52753–65. doi: 10.1021/acsami.4c13197 (PMC11450721; doi:10.1021/acsami.4c13197)
Supplement: Supplementary file 1 — am4c13197_si_001.pdf [file am4c13197_si_001.pdf]

# Supporting Information

## Interfacial Stabilization of Organic Electrochemical Transistors Conferred Using Polythiophene-Based Conjugated Block Copolymers with a Hydrophobic Coil Design

*Chia-Ying Li* [a], *Guo-Hao Jiang* [a], *Tomoya Higashihara* [b*], *Yan-Cheng Lin* [a,c*]

[a] Department of Chemical Engineering, National Cheng Kung University, Tainan 70101, Taiwan.

[b] Department of Organic Materials Science, Graduate School of Organic Materials Science, Yamagata University, 4-3-16 Jonan, Yonezawa, Yamagata 992-8510, Japan.

[c] Advanced Research Center for Green Materials Science and Technology, National Taiwan University, Taipei 10617, Taiwan.

[*] E-mail: thigashihara@yz.yamagata-u.ac.jp (T. Higashihara); ycl@gs.ncku.edu.tw (Y.-C. Lin)

**Table S1**. Optical and electrochemical properties of the polymers studied.

| Polymer | $\lambda_{onset}$ (nm) | $E_{g,opt}$ (eV) | $E_{onset,org}$ (V) | HOMO (eV) | LUMO (eV) | $E_{onset,aq}$ (V) | $E_{onset,aq} - E_{onset,org}$ (V) |
|---|---|---|---|---|---|---|---|
| P3HT | 647 | 1.92 | 0.44 | −5.20 | −3.28 | 0.71 | 0.27 |
| P3HT-*b*-PBA | 643 | 1.93 | 0.45 | −5.21 | −3.28 | 0.62 | 0.17 |
| P3HT-*b*-PS | 649 | 1.91 | 0.44 | −5.20 | −3.29 | 0.76 | 0.32 |
| P3HT-*b*-PEO | 645 | 1.92 | 0.40 | −5.17 | −3.25 | 0.63 | 0.23 |

**Table S2**. Summary of the reported P3HT for OECT application and their device parameters.

| **Materials** | ***W*/*L* (μm)** | **Electrolyte** | ***d* (nm)** | **$g_{m,norm}$ (S cm$^{-1}$)** | **$V_{th}$ (V)** | **$\mu C^*$ (F cm$^{-1}$ V$^{-1}$ s$^{-1}$)** | **Ref.** |
|---|---|---|---|---|---|---|---|
| P3HT | 200/20 | 0.1 M $KCl_{(aq)}$ | 80 | 12.5 | N/A | 10.4 | [1] |
| | 100/10 | 0.1 M $KCl_{(aq)}$ | 50 | 60.4 | −0.76 | 147 | [2] |
| | 3000/90 | EMIM:TFSI | 70 | N/A | −0.86 | 21.7 | [3] |
| | 10/5 | 0.1 M $NaCl_{(aq)}$ | 50 | 1.09 | −0.62 | 7.6 | [4] |
| | 1000/50 | 0.1 M $KCl_{(aq)}$ | 50 | 25.0 | −0.60 | N/A | [5] |
| P3HT | 9000/25 | 0.1 M $KCl_{(aq)}$ | 93 | 7.55 | −0.93 | 58.2 | This work |
| P3HT-*b*-PBA | | | 69 | 10.4 | −0.87 | 170 | |

**Table S3**. Summary of the mobility derived from (i) the slope of $g_m$ vs. $WdL^{-1}(V_{th} - V_g)$, (ii) transient gate current analysis at the time domain, and (iii) carrier density analysis.

| **Polymer** | $\mu$ (cm² s⁻¹ V⁻¹)[a] | $\mu C^*$ (F s⁻¹ cm⁻¹ V⁻¹)[a] | $\mu_\tau$ (cm² s⁻¹ V⁻¹)[b] | $\mu_\tau C^*$ (F s⁻¹ cm⁻¹ V⁻¹)[b] | $\mu_p$ (cm² s⁻¹ V⁻¹)[c] | $\mu_p C^*$ (F s⁻¹ cm⁻¹ V⁻¹)[c] |
|---|---|---|---|---|---|---|
| P3HT | 0.529 | 58.2 | $2.79 \times 10^{-3}$ | 0.307 | $4.17 \times 10^{-4}$ | 0.046 |
| P3HT-*b*-PBA | 0.518 | 170 | $6.13 \times 10^{-3}$ | 2.010 | $2.84 \times 10^{-3}$ | 0.931 |
| P3HT-*b*-PS | 0.084 | 38.3 | $5.53 \times 10^{-3}$ | 2.511 | $7.28 \times 10^{-4}$ | 0.330 |
| P3HT-*b*-PEO | 0.068 | 4.83 | $1.04 \times 10^{-4}$ | 0.007 | $8.49 \times 10^{-5}$ | 0.006 |

Mobility derived by [a] the slope of $g_m$ vs. $WdL^{-1}(V_{th} - V_g)$, [b] reciprocal transit time $-1/\tau_e$, and [c] carrier density $p$

**Table S4**. Summary of the coherence length ($L_c$) and relative degree of crystallinity (rDOC) of lamellar stacking and $\pi$–$\pi$ stacking of P3HT and BCPs.

| Polymer | Status | $L_c$(100) (Å)[a] | $L_c$(010) (Å)[b] | rDOC[c] |
|---|---|---|---|---|
| P3HT | As-cast | 84.3 | 46.3 | 1.00 |
| | Swelled | 80.8 | 23.7 | 0.83 |
| P3HT-*b*-PBA | As-cast | 98.6 | 32.3 | 0.78 |
| | Swelled | 100.9 | 33.7 | 0.88 |
| P3HT-*b*-PS | As-cast | 71.3 | 36.0 | 0.82 |
| | Swelled | 69.7 | 22.3 | 0.39 |
| P3HT-*b*-PEO | As-cast | 135.4 | 49.5 | 0.58 |
| | Swelled | 136.4 | 24.9 | 0.64 |

[a] Coherence length of lamellar stacking obtained by using Scherrer equation ($L_c = 2\pi K/\text{FWHM}$, $K = 0.9$). [b] Coherence length of $\pi$–$\pi$ stacking. [c] Relative degree of crystallinity determined by the ratio of integrated area in the geometrically corrected pole figure relative to P3HT.

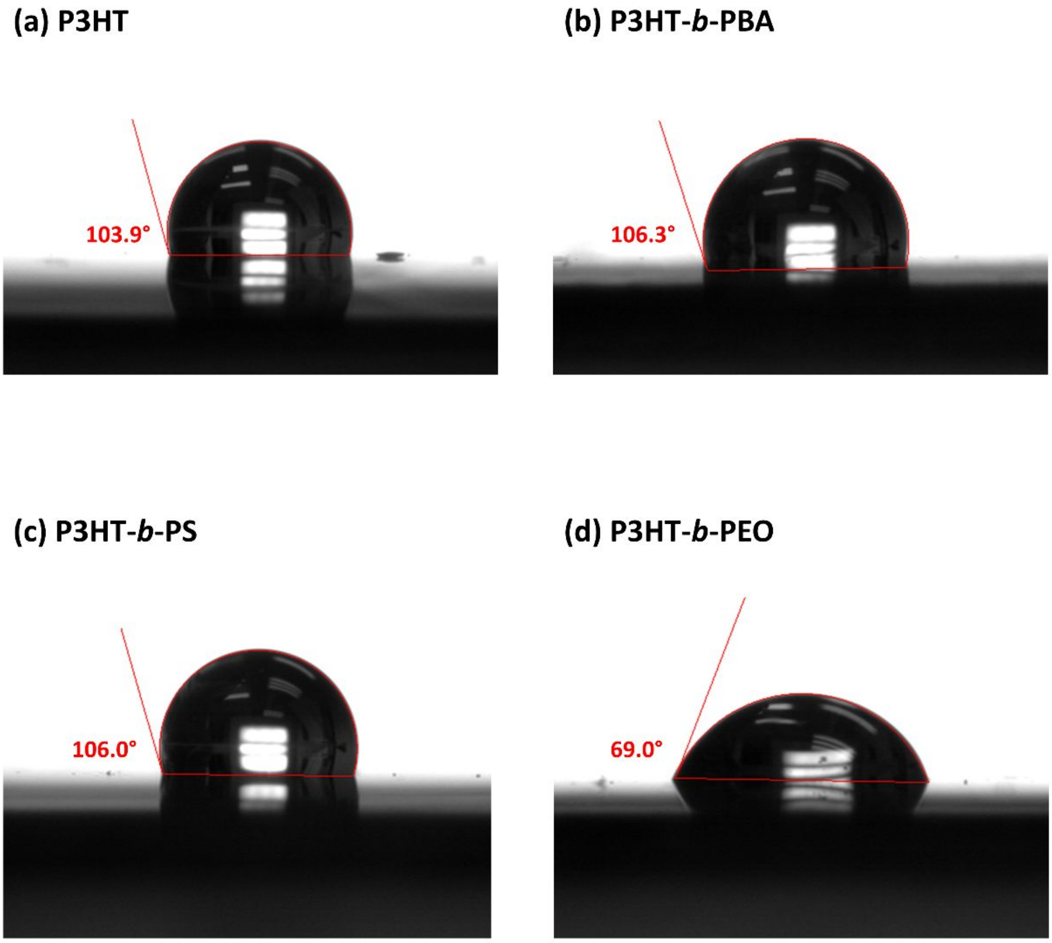


**Figure S1**. Contact angles of (a) P3HT, (b) P3HT-*b*-PBA, (c) P3HT-*b*-PS, and (d) P3HT-*b*-PEO.

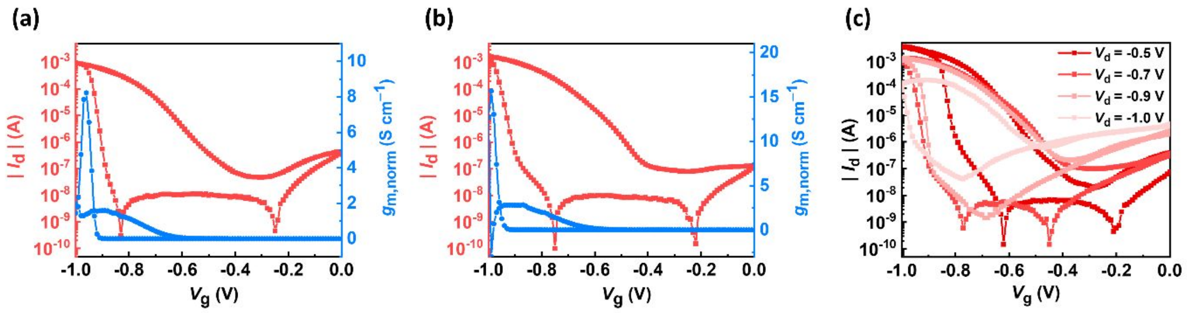


**Figure S2.** Transfer curves of P3HT-*b*-PBA based OECT devices in 0.1 M $KCl_{(aq)}$ with $V_d$ = −0.5 V and forward $V_g$ swept from 0 to −1.0 V. (a) with P3HT-*b*-PBA annealed at 150℃ for 30 minutes. (b) with bubbling nitrogen in $KCl_{(aq)}$ to remove oxygen. (c) Transfer curves with varying drain voltage applied.

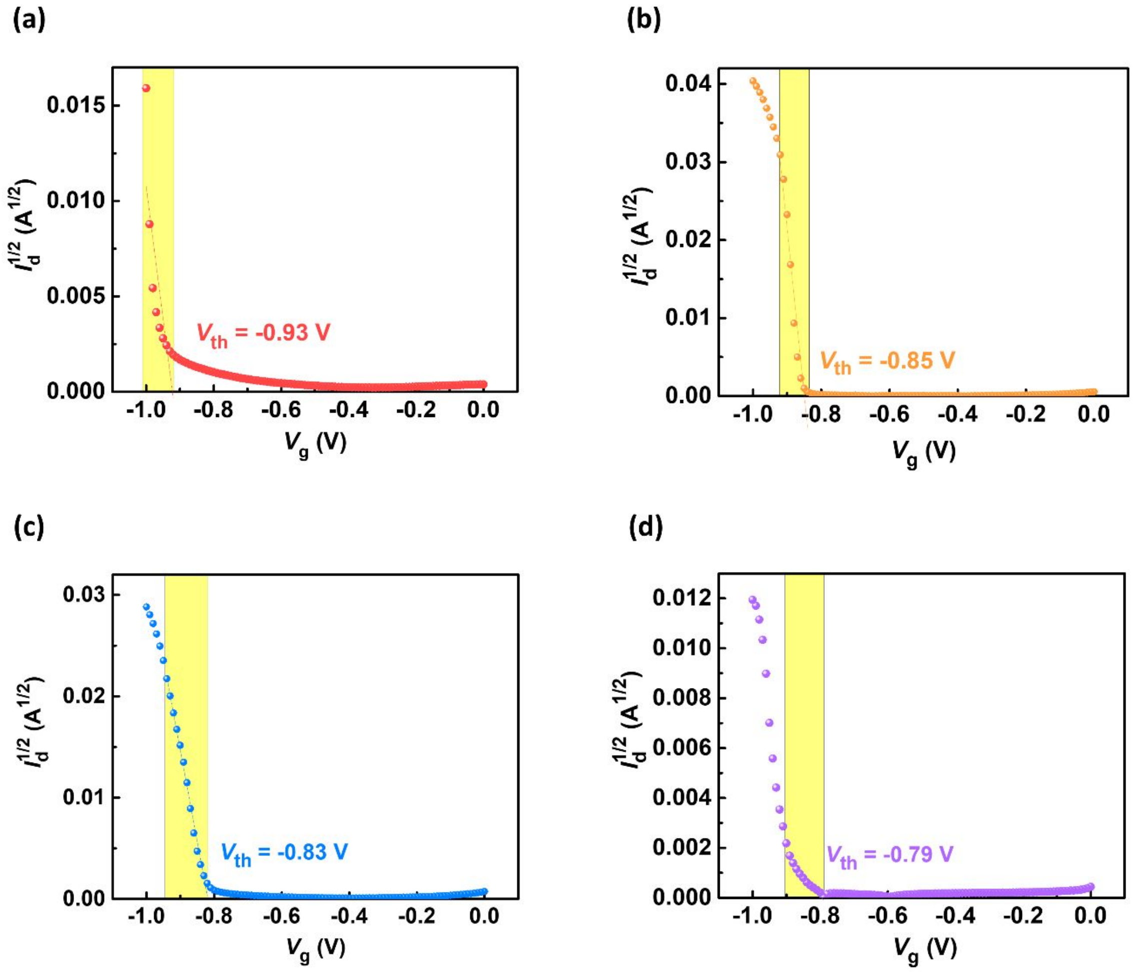


**Figure S3**. The relationship of $I_d^{1/2}$ vs. $V_g$ of (a) P3HT, (b) P3HT-*b*-PBA, (c) P3HT-*b*-PS, and (d) P3HT-*b*-PEO based OECT, including the linear fitting to determine the $V_{th}$ values.

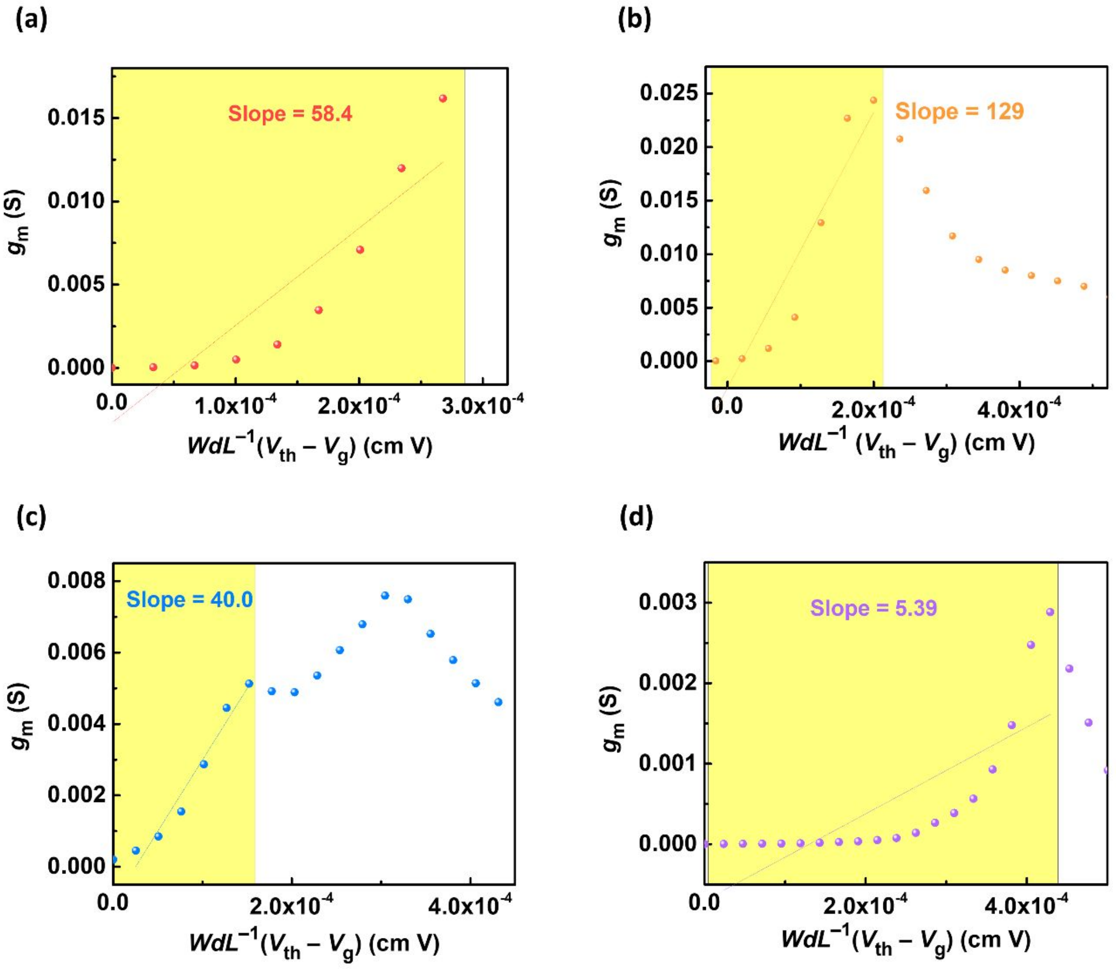


**Figure S4**. The relationship of $g_m$ vs. $WdL^{-1}(V_{th} - V_g)$ of (a) P3HT, (b) P3HT-*b*-PBA, (c) P3HT-*b*-PS, and (d) P3HT-*b*-PEO based OECT, including the linear fitting to determine the $\mu C^*$ values.

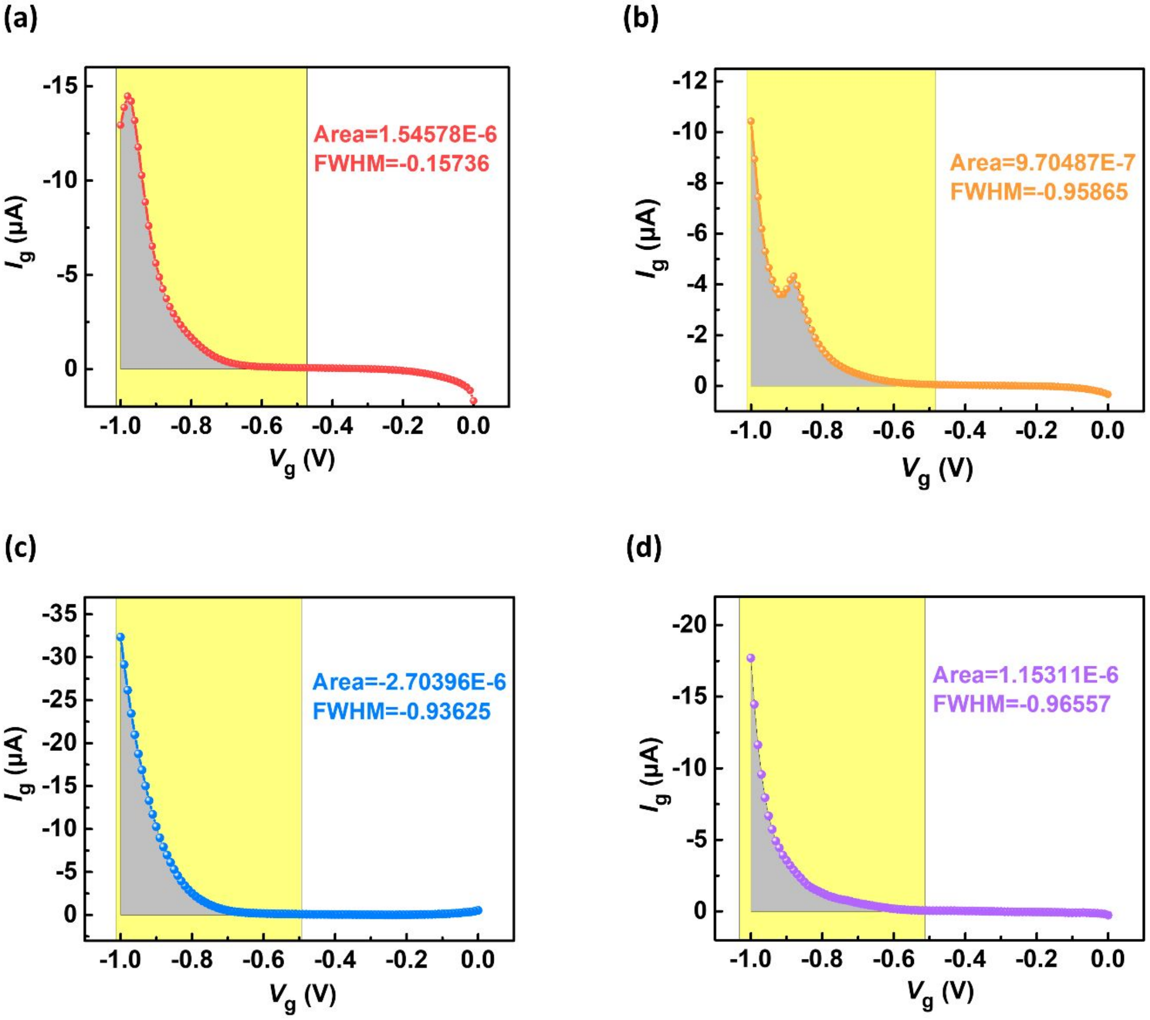


**Figure S5**. $I_g$ vs. $V_g$ plots of the (a) P3HT, (b) P3HT-*b*-PBA, (c) P3HT-*b*-PS and (d) P3HT-*b*-PEO based OECT, including the integrating area to determine the *p* values.

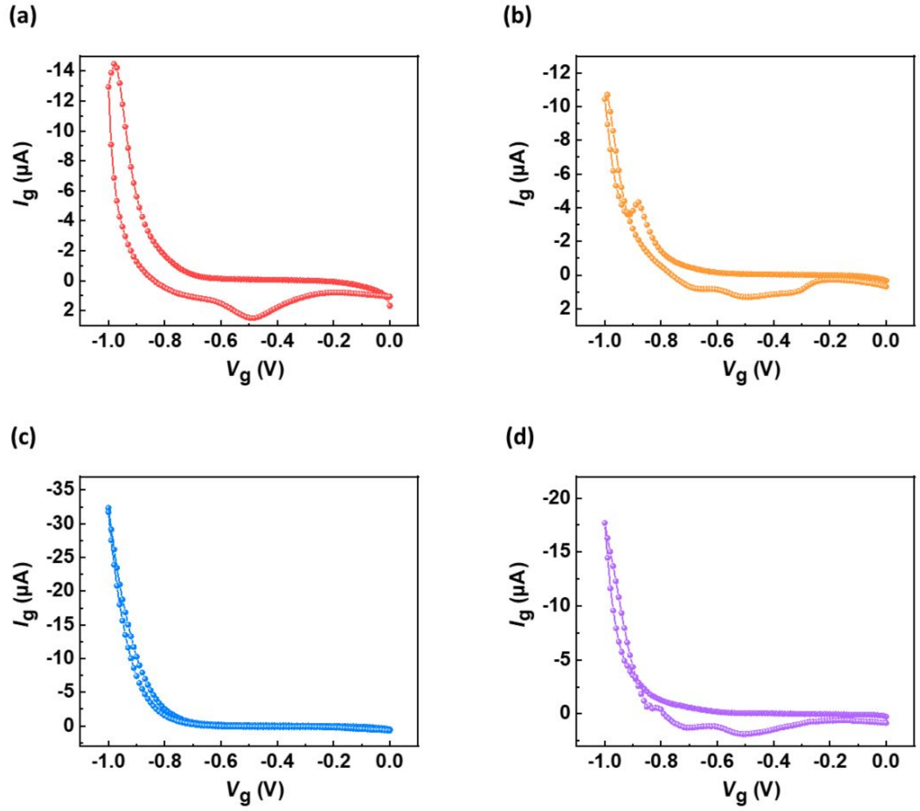


**Figure S6.** $I_g$ vs. $V_g$ plots of the (a) P3HT, (b) P3HT-*b*-PBA, (c) P3HT-*b*-PS and (d) P3HT-*b*-PEO based OECT in 0.1 M $KCl_{(aq)}$ including the reverse scan to describe their electrochemical behavior in the operating OECTs.

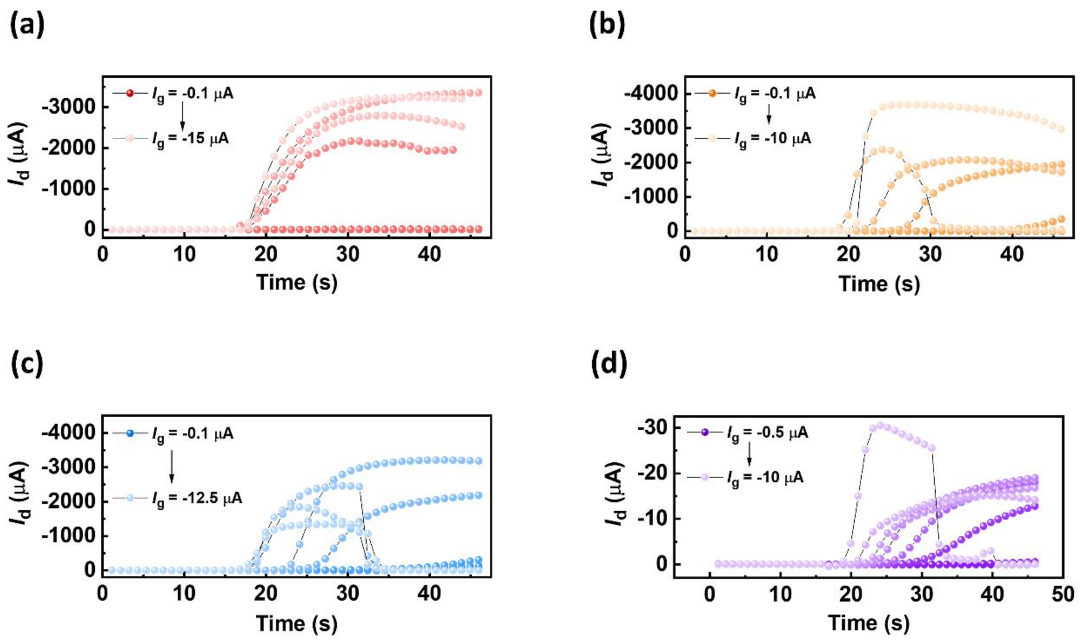


**Figure S7.** Transient response of (a) P3HT, (b) P3HT-*b*-PBA, (c) P3HT-*b*-PS, and (d) P3HT-*b*-PEO based OECT in 0.1 M $KCl_{(aq)}$ under application of a constant gate current, with $V_d$ = −0.5 V. In the first 15 seconds, there was no gate current applied ($I_g$ = 0 A), and a gate current was applied for 30 seconds. After that, a positive gate current was applied to dedope the polymer.

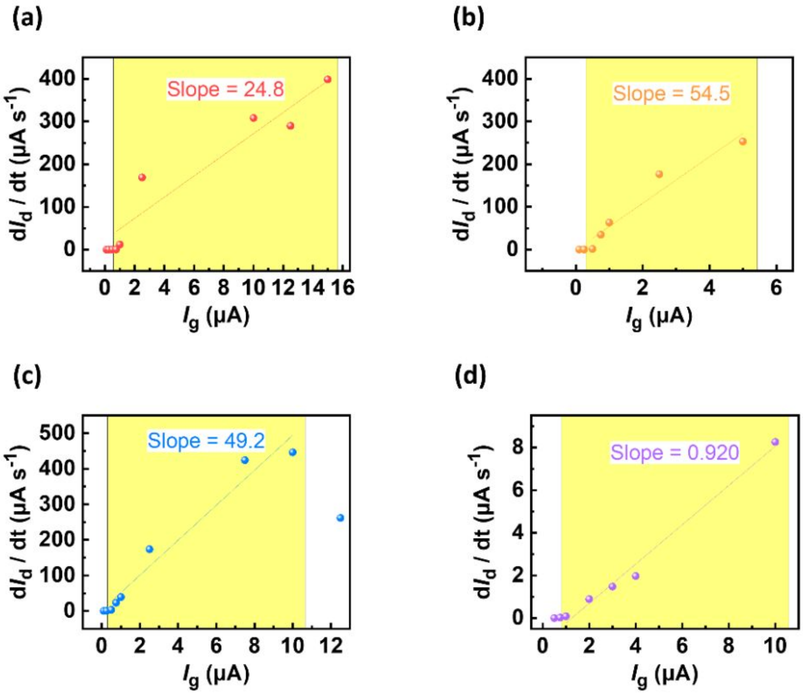


**Figure S8.** The relationship of $dI_d/dt$ vs. $I_g$ of (a) P3HT, (b) P3HT-*b*-PBA, (c) P3HT-*b*-PS, and (d) P3HT-*b*-PEO based OECT, including the linear fitting to determine the $-1/\tau_e$ values.

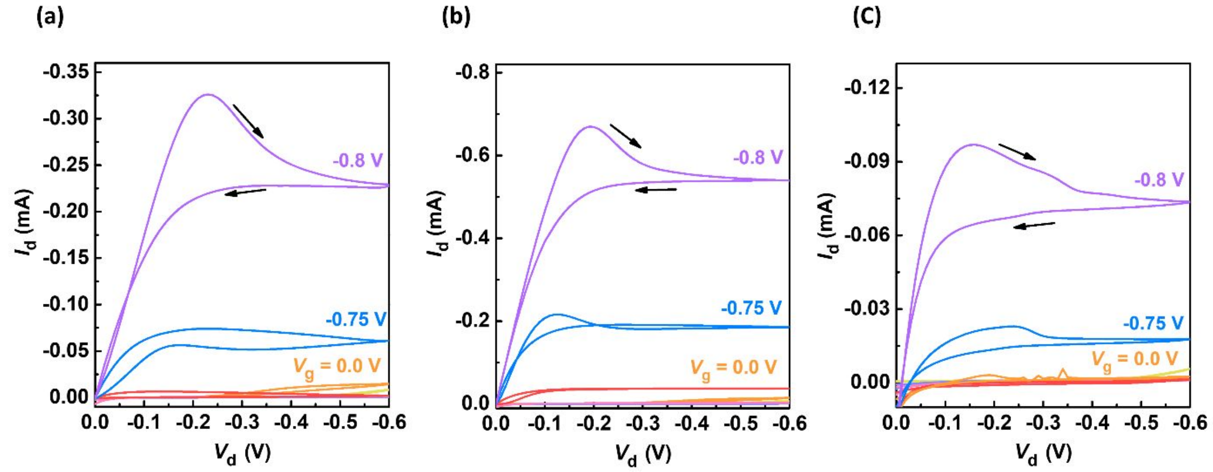


**Figure S9**. Output characteristics of the (a) P3HT, (b) P3HT-*b*-PS, and (c) P3HT-*b*-PEO based OECT devices.

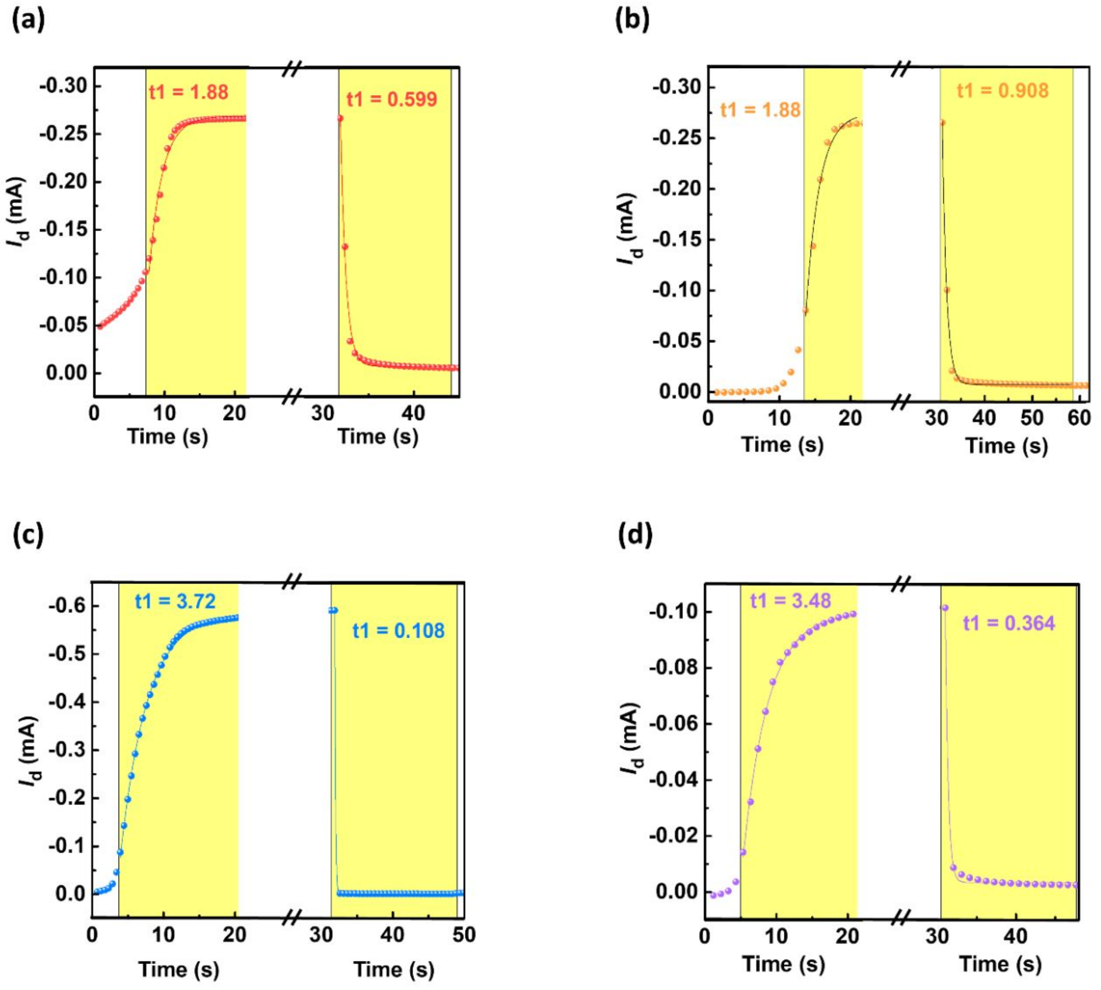


**Figure S10**. Transient curves of the (a) P3HT, (b) P3HT-*b*-PBA, (c) P3HT-*b*-PS, and (d) P3HT-*b*-PEO based OECT, including the exponential fitting to determine the $t_r$ and $t_f$ values.

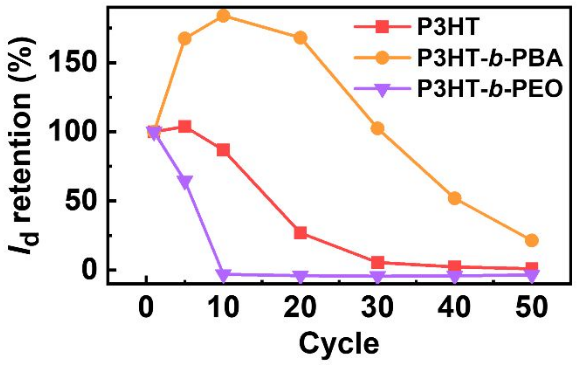


**Figure S11.** Comparison of $I_d$ retention of the P3HT, P3HT-*b*-PBA, and P3HT-*b*-PEO for 50 cycles at $V_d = -0.1$ V and $V_g$ switched between 0 and −0.9 V.

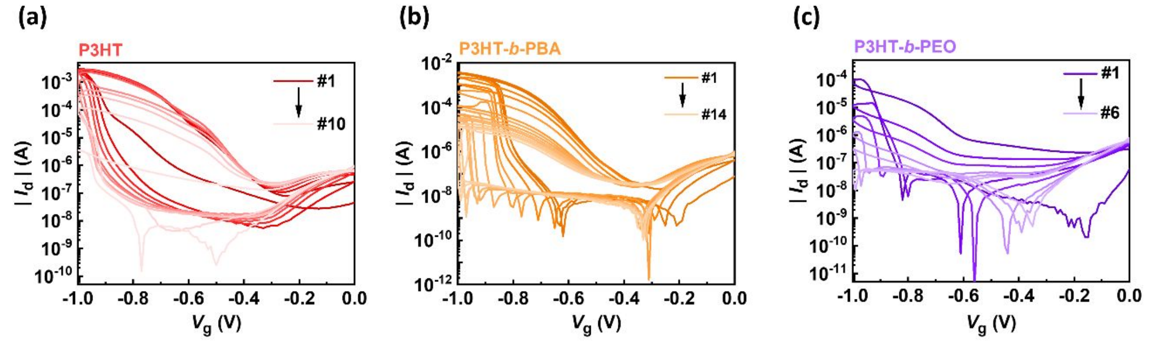


**Figure S12.** Multiple cycles of transfer curves for (a) P3HT, (b) P3HT-*b*-PBA, and (c) P3HT-*b*-PEO based OECT devices in 0.1 M $KCl_{(aq)}$ with $V_d = -0.5$ V and forward $V_g$ swept from 0 to −1.0 V.

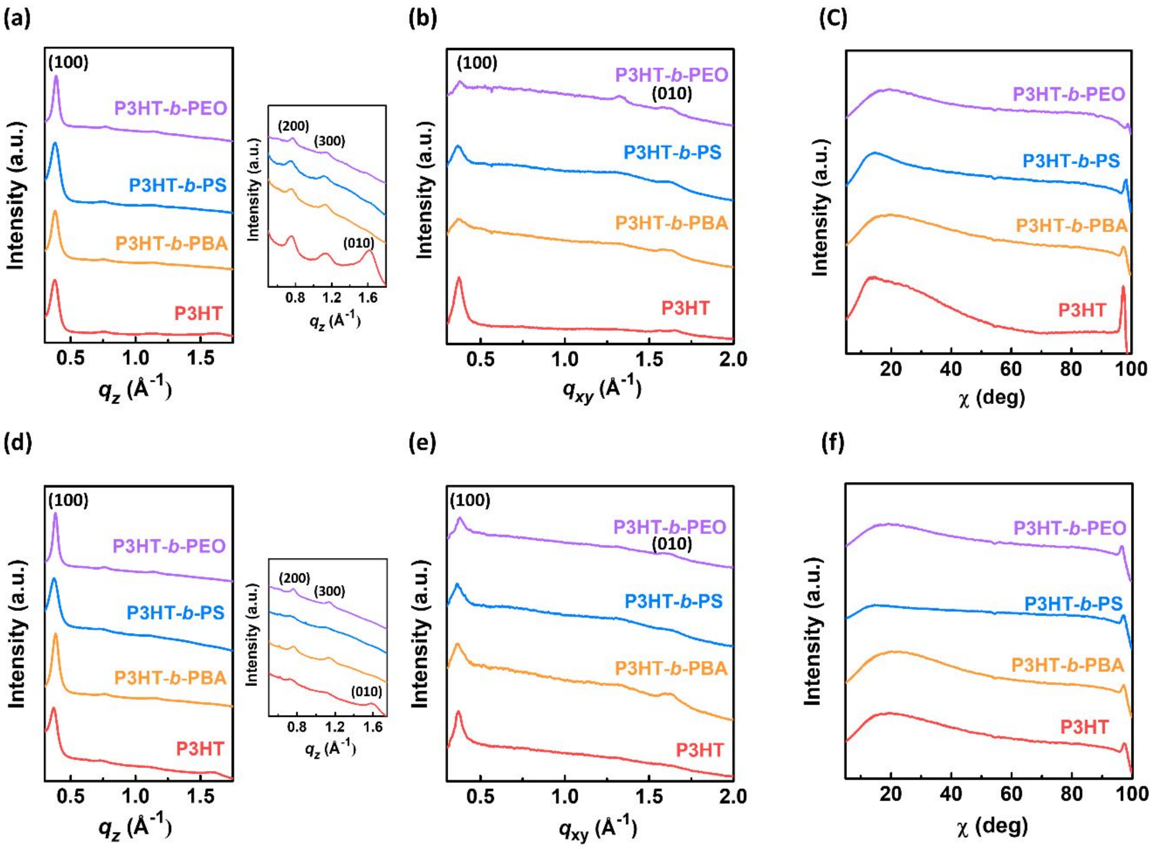


**Figure S13**. 1D GIXD profile in the (a,d) out-of-plane and (b,e) in-plane directions and (c,f) the geometrically corrected pole figure of (a–c) the as-cast and (d–f) the electrolyte-swelled BCP films.

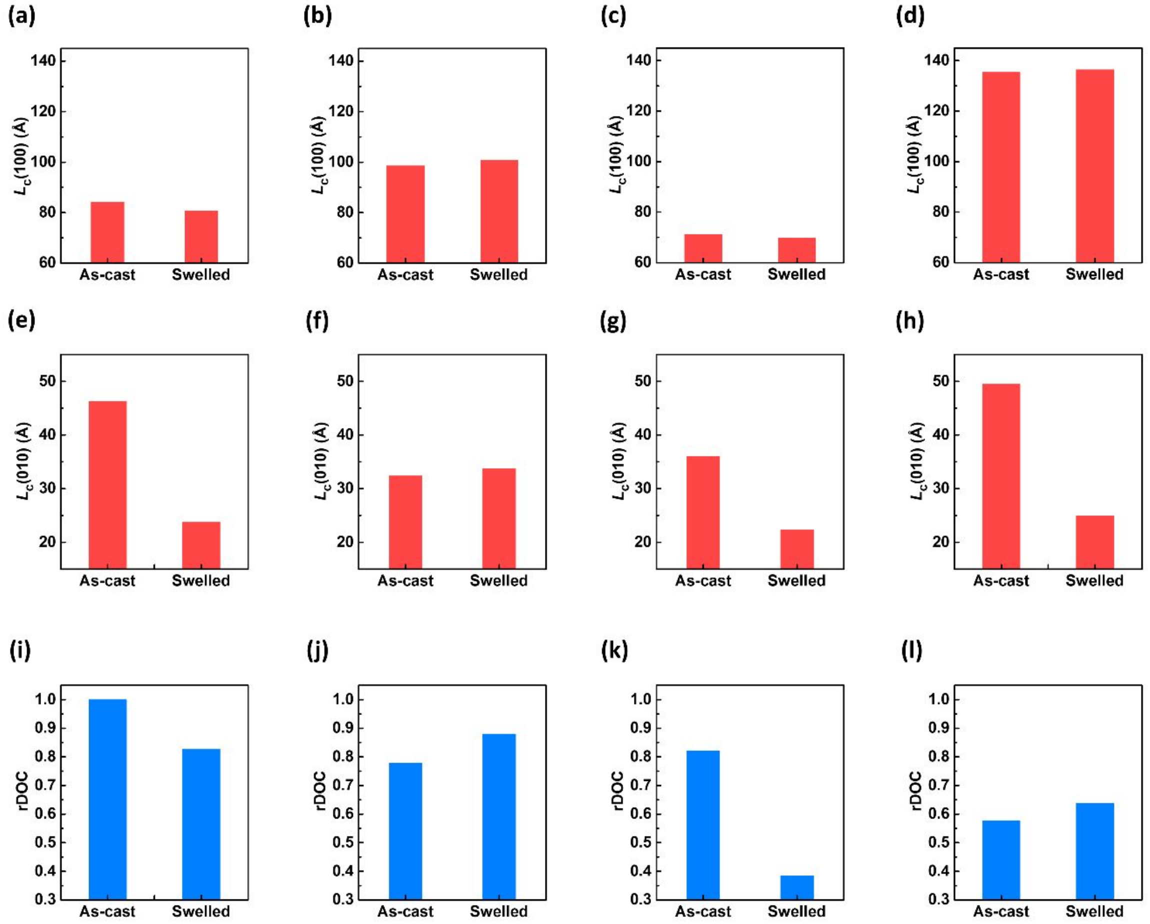


**Figure S14**. Comparison of the crystallographic parameters with (a–d) lamellar (100) and (e–h) $\pi$–$\pi$ stacking (010) coherence length ($L_c$), and (i–l) relative degree of crystallinity for (a,e,i) P3HT, (b,f,j) P3HT-*b*-PBA, (c,g,k) P3HT-*b*-PS, and (d,h,l) P3HT-*b*-PEO.

**REFERENCES**

(1) Flagg, L. Q.; Bischak, C. G.; Onorato, J. W.; Rashid, R. B.; Luscombe, C. K.; Ginger, D. S. Polymer Crystallinity Controls Water Uptake in Glycol Side-Chain Polymer Organic Electrochemical Transistors. *J. Am. Chem. Soc.* **2019**, *141* (10), 4345-4354.

(2) Huang, L.; Wang, Z.; Chen, J.; Wang, B.; Chen, Y.; Huang, W.; Chi, L.; Marks, T. J.; Facchetti, A. Porous Semiconducting Polymers Enable High-Performance Electrochemical Transistors. *Adv. Mater.* **2021**, *33* (14), 2007041.

(3) Yadav, Y.; Ghosh, S. K.; Singh, S. P. High-Performance Organic Field-Effect Transistors Gated by Imidazolium-Based Ionic Liquids. *ACS Appl. Electron. Mater.* **2021**, *3* (3), 1496-1504.

(4) Kim, H. J.; Perera, K.; Liang, Z.; Bowen, B.; Mei, J.; Boudouris, B. W. Radical Polymer-Based Organic Electrochemical Transistors. *ACS Macro Lett.* **2022**, *11* (2), 243-250.

(5) Teng, X.; Sun, J.; Jiang, J.; Ke, S.; Li, J.; Lou, Z.; Hou, Y.; Hu, Y.; Teng, F. Ion effects on salt-in-water electrolyte gated polymer electrochemical transistors. *Org. Electron.* **2023**, *120*, 106859.
